# Early Identification of Alzheimer's Disease in Mouse Models: Application of Deep Neural Network Algorithm to Cognitive Behavioral Parameters

**DOI:** 10.1016/j.isci.2021.102198

**Published:** 2021-02-16

**Authors:** Stephanie Sutoko, Akira Masuda, Akihiko Kandori, Hiroki Sasaguri, Takashi Saito, Takaomi C. Saido, Tsukasa Funane

**Affiliations:** 1 Hitachi, Ltd, Research and Development Group, Center for Exploratory Research, Kokubunji, Tokyo 185-8601, Japan; 2 Laboratory for Proteolytic Neuroscience, RIKEN Center for Brain Science, Wako, Saitama 351-0198, Japan; 3 Organization for Research Initiatives and Development, Doshisha University, Kyotanabe, Kyoto 610-0394, Japan; 4 Department of Neurocognitive Science, Institute of Brain Science, Nagoya City University Graduate School of Medical Sciences, Nagoya, Aichi 467-8601, Japan

**Keywords:** systems neuroscience, cognitive neuroscience, systems biology, model organism

## Abstract

Alzheimer's disease (AD) is a worldwide burden. Diagnosis is complicated by the fact that AD is asymptomatic at an early stage. Studies using AD-modeled animals offer important and useful insights. Here, we classified mice with a high risk of AD at a preclinical stage by using only their behaviors. Wild-type and knock-in AD-modeled (*App*^*NL-G-F/NL-G-F*^) mice were raised, and their cognitive behaviors were assessed in an automated monitoring system. The classification utilized a machine learning method, i.e., a deep neural network, together with optimized stepwise feature selection and cross-validation. The AD risk could be identified on the basis of compulsive and learning behaviors (89.3% ± 9.8% accuracy) shown by AD-modeled mice in the early age (i.e., 8–12 months old) when the AD symptomatic cognitions were relatively underdeveloped. This finding reveals the advantage of machine learning in unveiling the importance of compulsive and learning behaviors for early AD diagnosis in mice.

## Highlights

• Cognitive-related behaviors were monitored to identify Alzheimer's disease (AD) mice
• Those behaviors were used as inputs of the deep neural network algorithm
• Best-performing inputs were related to age-specific mild decreases of cognitions
• Despite underdeveloped symptoms, AD mice could be distinguished from the early age

## Graphical abstract

**Figure:**
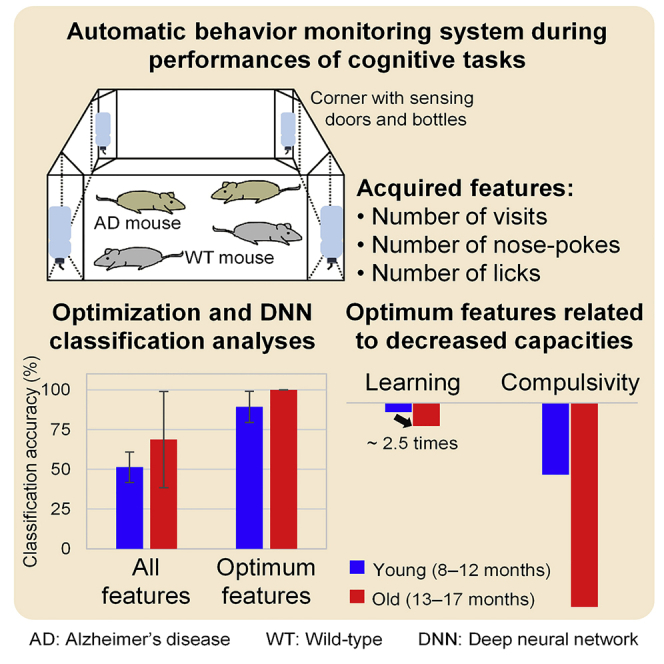

## Introduction

Dementia has been a worldwide concern. The prevalence of dementia is predicted to increase by more than twice by 2030 and even thrice by 2050 with greater prevalence in low- and middle-income countries (World Health Organization and Alzheimer’s Disease, 2012; Prince et al., 2016). Alzheimer's disease (AD) is the most common type of dementia, accounting for 60%–70% of dementia cases (World Health Organization and Alzheimer’s Disease, 2012). The onset of AD varies, starting from 65 years old (late-onset AD), but the age range with the highest probability of onset is 85 years and older (Qiu et al., 2009). Core symptoms involve memory decline (e.g., in spatial, semantic, implicit, episodic memory), impaired speech and linguistic abilities, and problems with executive functions, whereas other behavioral and psychological symptoms of dementia such as depression, personality changes (e.g., irritation, aggression), presence of hallucinations, and delusional thinking also frequently occur in patients with AD. These symptomatic behaviors seriously disrupt daily activities and lower the quality of life. Between 2000 and 2010, the mortality rate of AD increased by up to 38.7% in the United States (Tejada-Vera, 2013), and it is currently the sixth leading cause of death (Xu et al., 2016). The global economic cost, including direct medical, direct social, and informal care costs, was estimated at US$ 604 billion in 2010 (Wimo et al., 2013) and is expected to increase in the near future (Prince et al., 2016). In Europe, the economic cost was estimated at €105.2 billion in 2011 (Smith, 2011). This cost burden is comparable to the gross domestic product of large countries (e.g., Turkey and Indonesia) (Wortmann, 2012).

The late stage of AD characterized by neuronal death is irreversible. Currently, two types of antidementia drug, acetylcholinesterase inhibitors and an N-methyl-D-aspartate (NMDA) receptor antagonist, are available in clinical practice. In addition, drugs targeting amyloid-β peptide (Aβ), such as anti-Aβ antibodies and β-secretase 1 inhibitors, are under clinical trials. Although there is still a debate as to whether these antidementia drugs are able to decelerate disease progression (Rountree et al., 2009) or only relieve symptoms (Rountree et al., 2012; Szeto and Lewis, 2016), early detection of those at high risk of AD is essential (Cummings et al., 2007; Petersen, 2009). Longitudinal data (e.g., neuroimaging, biochemical, and genetic data) have started to be collected in projects aimed at the prevention and treatment of AD, such as the Alzheimer's disease neuroimaging initiative (ADNI); the Australian imaging, biomarkers & lifestyle flagship study of aging (AIBL); Japanese ADNI; and European ADNI (Hendrix et al., 2015). The deposition of Aβ in the brains of patients with subclinical cognitive impairment has been proposed as a predictor of AD risk (Morris et al., 2009; Berti et al., 2010; Landau et al., 2012). However, Aβ deposition may also be detected in the brains of cognitively normal elderly people (Driscoll and Troncoso, 2011). Thirty to fifty percent of deceased elderly people who had undergone postmortem examination and showed the presence of Aβ deposition were reported to be clinically normal individuals (O'Brien et al., 2009; Price et al., 2009). Therefore, the AD pathophysiological hypothesis related to Aβ deposition is still confounding.

The progression from Aβ deposition to visible clinical symptoms may take more than a decade to confirm (Jack et al., 2009, 2010). This time lag is influenced by high interindividual variability caused by differences in genetic backgrounds (Kunkle et al., 2019) and in brain and cognitive reserve and pathological comorbidity (Jack et al., 2010; Sperling et al., 2011). Prolonged and varying time lag complicates the studies of AD diagnosis, screening biomarkers, disease mechanisms, and therapeutic development. Here, animal research has been a common approach to accelerating these studies. A hypothesis of AD pathophysiology is neuroinflammation mediated by microglia and astrocytes causing cerebral Aβ plaque and behavior-cognitive impairments. A mouse model was created by using a knock-in (KI) strategy to mimic Aβ overproduction (Saito et al., 2014; Sasaguri et al., 2017). Another common method is AD-modeled mice based on the amyloid precursor protein (APP)-overexpressing approach. Both methods produce AD-like phenotypes with different Aβ deposition rates and severity symptoms. Some artifacts of the APP-overexpressing model, such as non-specific overproduction of APP fragment proteins (Hsiao et al., 1996; Mucke et al., 2000), abnormal gene expressions (Goodwin et al., 2019), and destroyed exons of fibroblast growth factor 14 (Gamache et al., 2019), have been found. Despite these artifacts, APP-overexpressing mice are able to project moderate to severe behavioral phenotypes. Meanwhile, *App* KI mice show only preclinical AD-relevant phenotypes without any associations with APP-overexpressing mice. Therefore, the use of specific AD-modeled mice should be engineered according to the purposes of the study (Sasaguri et al., 2017).

Even though Aβ deposition may bring important insights, a quantitative measure of it using positron emission tomography (PET) is relatively expensive and invasive due to the binding-tracer injection. Without routine measurement, the onset of Aβ deposition is sometimes overlooked. Cognitive declines and behavioral changes hence become significant but delayed symptoms. By studying AD-modeled mice, we hope to classify those at high risk of AD on the basis of only their behaviors at an early age (i.e., preclinical AD stage). For early AD screening, the implementation of the *App* KI model with preclinical AD phenotypes is suitable. A KI mouse model, namely, *App*^*NL-G-F/NL-G-F*^, has been reported to reveal an increase in Aβ deposition, broad neuroinflammation, and cognitive deficits (Masuda et al., 2016). *App*^*NL-G-F/NL-G-F*^ mice exhibit three mutations: Swedish (NL), Beyreuther/Iberian (F), and Arctic (G). In this study, the classification was supported by a machine learning algorithm, namely, a deep neural network (DNN). The usefulness of machine learning has been evidenced in predicting AD progression from mild cognitive impairment (MCI) using six data types: (1) PET patterns (Katako et al., 2018; Ding et al., 2019), (2) structural magnetic resonance imaging (MRI) patterns (Plant et al., 2010; Moradi et al., 2015; Long et al., 2017), (3) functional MRI (Hojjati et al., 2017), (4) socio-demographic information and clinical and neuropsychological scores (Clark et al., 2014, 2016; Johnson et al., 2014; Grassi et al., 2018), (5) blood plasma proteins (Agarwal et al., 2015), and (6) blood-based markers of cerebrospinal fluid Aβ (Goudey et al., 2019). Machine learning has been applied to numerous AD studies; the current study features easy-to-collect behavioral data of AD mouse models using an automated monitoring system. It addresses two major points: classification between genotypes of mice using only behaviors and identification of influential behaviors for classifying high-risk mice (i.e., *App*^*NL-G-F/NL-G-F*^).

## Results

### Automatic quantification of behavioral parameters

There is an extensive debate about the animal-human comparative psychology (Morgan, 1903; Zentall, 1999; Fitzpatrick, 2008; Meketa, 2014; Mercado, 2016; Starzak, 2017); interdisciplinary fields strive for the understanding of animal cognition. The comparison of animal-human cognitions show both similarities (e.g., conceptual understanding, spatial learning, memory, social mental, and imitation) (Kuhlmeier and Boysen, 2006) and discrepancies (e.g., teaching, memory capacity, causal reasoning, planning, deception, transitive inference, and theory of mind) (Premack, 2007). These discrepancies might be justifiable due to macroscopic and microscopic gaps (e.g., anatomy, size, neural structure, neural wiring and connectivity, etc.) between human and animal brains. Meanwhile, these cognitive similarities should consider species-dependent capacities.

During the test tasks (Figure S1), five tasks (Figure S2) were designed to evaluate cognitive behaviors, such as learning, impulsivity, attention, and compulsivity, in wild-type (WT) and *App*^*NL-G-F/NL-G-F*^ mice. To minimize the risks of over- and under-attribution biases, the cognitive interpretation from the currently performed tasks had been investigated and reported in the previous studies (D'Hooge and De Deyn, 2001; Ryan et al., 2013; Kiryk et al., 2020). The term of cognitive behavior is defined as specific responses from animals during task performances that likely represent cognitive functions. When the mice were performing the test tasks, their activities (numbers of visits to corner chambers, numbers of nose-pokes at doorways, and numbers of licking at drinking bottles) were automatically recorded (see Figure S3 for exemplary time series data). These activities were then defined as behavioral parameters. Some of these parameters represented behavioral performances in each cognitive task. For example, in the place preference learning test, mice were allowed to access water bottles only in a correct corner out of four corners, which was individually assigned to avoid learning imitation between mice. The number of correct visits suggests the spatial learning ability of recognizing the correct corner chambers. As some factors (e.g., motor functions, anxiety traits) other than corresponding cognitions possibly affect the behavioral parameters, we carefully checked that there was no impaired baseline activity (e.g., general visits and nose-pokes) in *App*^*NL-G-F/NL-G-F*^ mice before the performance of cognitive tasks (Masuda et al., 2016). The number of correct corner visits in the place preference reversal (PPR) learning test, where the correct corner shifted diagonally to the original position, quantified the ability to learn in changing circumstances (learning flexibility). In the serial reaction time test (SRTT), after a nose-poke, the mice had to wait (1.0, 2.0, or 4.0 s) for the doorway to open for a certain period (0.3, 0.5, or 1 s) with the cue being the light-emitting diode (LED) switching on. A large number of nose-pokes during the waiting period (i.e., premature visit/trial) indicated poor impulsivity control. The number of drinks (i.e., correct visits/trials) quantified the control of attention, i.e., how quickly the mice reacted after the LED was switched on and the doorway opened. Furthermore, in the place avoidance learning test, a nose-poke at the supposed-to-be-avoided corner chamber would trigger a puff of air (i.e., learning process). After the learning process, the avoidance condition was eliminated (i.e., no puff of air), and the number of nose-pokes at the previously avoided corner chamber quantified the retention (i.e., 24 h after the elimination of avoidance) and extinction (i.e., 5 days after the elimination of avoidance) learning. In the delay-discounting test, to access a 0.5% saccharin solution, the mice had to wait (1–8 s after a nose-poke) for the doorway to open. Instead of waiting a long time to drink the saccharin solution, the mice could choose to drink water from the adjacent bottle without waiting for the doorway to open (0 s after a nose-poke). Large numbers of nose-pokes and licks at the doorway of the saccharin solution represented compulsive/persistent behaviors. All test parameters, categorized in 11 parameter groups, are tabulated in Table 1.

**Table 1:** Summary of test parameters

Test	Class	Parameter and its description	Number of parameters
Place preference learning	1	Daily visit rate to the correct corner chamber (7 days)	7
Place preference reversal learning	2	Daily visit rate to the correct corner chamber (7 days)	7
Serial reaction time, impulsivity	3	Daily premature trial (i.e., did nose-poke(s) during the delay period) rate (10 days including training)^∗^	8
Serial reaction time, attention (days 5–7)	4	Premature trial rate at stimulus durations of 0.3, 0.5, and 1 s	3
Serial reaction time, attention (days 5–7)	5	Omission error (i.e., missed to drink at the open door) rate at stimulus durations of 0.3, 0.5, and 1 s	3
Serial reaction time, attention (days 5–7)	6	Omission trial (i.e., missed to initiate the test) rate at stimulus durations of 0.3, 0.5, and 1 s	3
Serial reaction time, attention (days 5–7)	7	Correct rate from total trial (i.e., visit) at stimulus durations of 0.3, 0.5, and 1 s	3
Serial reaction time, attention (days 5–7)	8	Correct rate from total non-premature trial (i.e., visit) at stimulus durations of 0.3, 0.5, and 1 s	3
Place avoidance learning	9	Nose-poke error rate at avoided doorways during baseline, learning, retention, and extinction periods	4
Delay-discounting	10	Nose-poke rate at doorways to access the saccharin solution with delays of 0, 0.1, 1, 2, 3, 4, 5, 6, 7, and 8 s^∗^	9
Delay-discounting	11	Lick rate at saccharin bottles with delays of 0, 0.1, 1, 2, 3, 4, 5, 6, 7, and 8 s	10

The test parameters (60 in total) were categorized into 11 parameter groups according to test type and parameter descriptions. Impulsivity and delay discounting tests (∗) had a reduced number of parameters because data were unavailable for more than half of the sample number. See also Figure S3, Table S1, and Table S2.

### *App*^*NL-G-F/NL-G-F*^ mice reveals impaired functions of compulsivity control, learning, and attention

**Figure 1:**
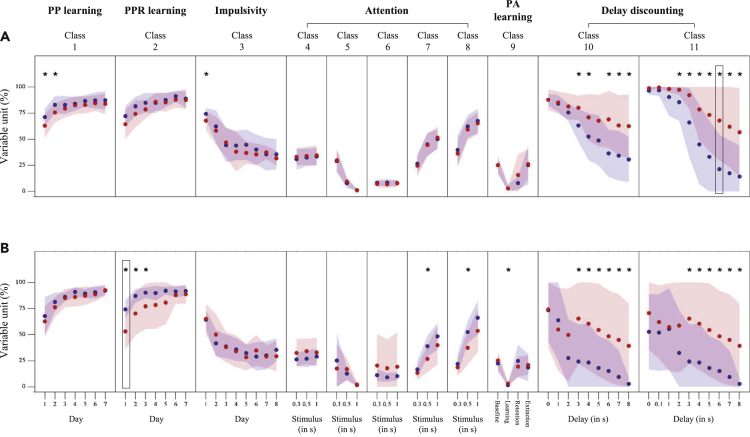
Behavioral parameters (A and B) This figure shows behavioral parameters in phases 1 (A) and 2 (B) for WT (blue plots) and *App*^*NL-G-F/NL-G-F*^ (red plots) mice. Scattered bullets represent averages of behavioral parameters, and patches around bullets indicate standard deviations of behavioral parameters. Asterisks (∗) denote parameters significantly showing different averages between WT and *App*^*NL-G-F/NL-G-F*^ mice (two-sample *t* test; p < 0.05). Black rectangles show starter features. See also Figures S1 and S2, Tables S1 and S2, and Data S1.

**Figure 2:**
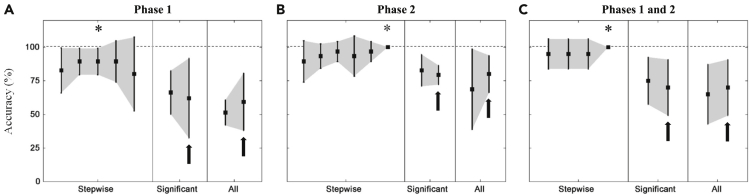
Classification performances of the DNN analysis (A–C) This figure shows classification performances (i.e., accuracy) of the DNN analysis using behavioral data of test tasks during phases 1 (A), 2 (B), and both (C) with different feature-selecting methods (stepwise feature, significant between-genotype parameters, and all parameters). Asterisks (∗) denote the optimum cross-validated accuracy for the stepwise selection method. Black arrows represent classification results with gender also included as an input node. Error bars represent standard deviations of cross-validated accuracies. See also Figure S4.

Figure 1 shows the behavioral parameters in phases 1 (age 8–12 months; Figure 1A) and 2 (age 13–17 months; Figure 1B) for each genotype. The starter features (black-lined squares in Figure 1) were found in parameter-groups 11 (6 s delay) and 2 (day 1) for phases 1 and 2, respectively. There are two points of highlight. First, the behavioral parameters of the delay-discounting test were significant between-genotype differences in phase 1 (two-sample *t* test; *t*_(25)_ = 2.14–3.38; p < 0.05). The *App*^*NL-G-F/NL-G-F*^ mice developed a more severe compulsion toward the saccharin solution than the WT mice did. Instead of waiting (>2 s) to access the saccharin solution, the WT mice chose to drink from water bottles. Therefore, their nose-poke and lick rates were significantly decreased compared with those of the *App*^*NL-G-F/NL-G-F*^ mice. These between-genotype differences were consistent with those in phase 2 (two-sample *t* test; *t*_(27)_ = 2.38–3.33; p < 0.05). Second, between-genotype differences were more frequently found in phase 2. For example, during the PPR learning test, the *App*^*NL-G-F/NL-G-F*^ mice made significantly more mistakes than the WT mice (two-sample *t* test; *t*_(27)_ = 2.17–4.15; p < 0.05), as indicated by the low visit rate to the correct corner chamber (parameter-group 2) in phase 2 (Figure 1B) compared with in phase 1 (Figure 1A; two-sample *t* test; *t*_(25)_ = 0.29–1.74; p > 0.05). Furthermore, the WT mice performed well with a higher correct rate (parameter-groups 7 and 8) when responding to the opened doorways (0.5 s) during the SRTT than that of the *App*^*NL-G-F/NL-G-F*^ mice (two-sample *t* test; *t*_(27)_ = 2.84–2.93; p < 0.05). There were significant between-genotype differences in phase 1 but not in phase 2; however, there was no significant inter-phase (i.e., phases 1 versus 2; parameter-group 1 for days 1 and 2) difference in either genotype (one-sample *t* test; *t*_(17)_ = 0.35–1.19 for WT; *t*_(13)_ = 0.41–0.96 for *App*^*NL-G-F/NL-G-F*^; p > 0.05). Genotype-dependent aging might suggest a reason for this difference in significance (inter-phase parameter-group 3 of day 1; one-sample *t* test; *t*_(17)_ = 3.97 for WT; *t*_(13)_ = 0.27 for *App*^*NL-G-F/NL-G-F*^; only significant for the WT mice). In summary, the cognitive impairments were reflected early in the lack of control of compulsive behaviors and were followed by declining learning flexibility and attention.

### Stepwise approach optimizes feature selection for high classification performance

All behavioral parameters were potentially used as classifying features in the DNN algorithm. The effects of the feature-selecting methods and phases were compared in terms of classification performance, as shown in Figure 2. The numbers of significant between-genotype features were 15 (phase 1), 18 (phase 2), and 20 (both phases). Accordingly, it was difficult to distinguish the benefits of the significant between-genotype method from those of the all parameters method. Using all parameters of phase 1 was worse than using significant between-genotype parameters (Figure 2A). Meanwhile, classification performances were relatively unchanged by using either significant between-genotype or all parameters of phase 2 and both phases (Figures 2B and 2C). The use of gender as an input node (black arrows in Figure 2) may not improve performance. Among the feature-selecting methods, the stepwise method (Figure S4) gave the highest classification performance. Even from the behavioral parameters of the earliest phase (i.e., phase 1), the WT and *App*^*NL-G-F/NL-G-F*^ mice could be distinguished with 89.3% ± 9.8% accuracy. It has been reported that *App*^*NL-G-F/NL-G-F*^ mice aged 8–9 months showed only a few of the behavioral dysfunctions (i.e., preclinical behaviors) (Hamaguchi et al., 2019); yet, deposition of pathological Aβ and gliosis have also been reported (Masuda et al., 2016). The high classification accuracy suggests the prospect of early detection for AD risk by using only behavioral information. Therefore, behavioral parameters of the later phase (i.e., phase 2) with more developed symptoms resulted in perfect classification (100%).

### Benefits of DNN algorithm

**Figure 3:**
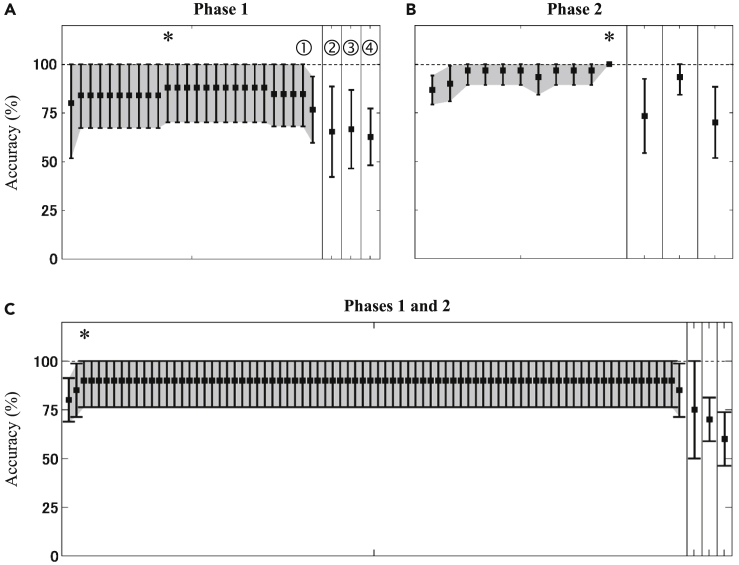
Classification performances of the control analysis (A–C) This figure shows classification performances (i.e., accuracy) of the control analysis using behavioral data of test tasks during phases 1 (A), 2 (B), and both (C) with different feature-selecting methods (stepwise feature [➀], significant between-genotype parameters [➁ and ➂ for *App*^*NL-G-F/NL-G-F*^ > WT and *App*^*NL-G-F/NL-G-F*^ < WT, respectively], and all parameters [➃]). Asterisks (∗) denote the optimum cross-validated accuracy for the stepwise selection method. Error bars represent standard deviations of cross-validated accuracies.

Before the implementation of machine learning algorithms, the classification computation was performed by following the conventional threshold approach in which the classifying features were evaluated on the basis of the thresholds (e.g., features > thresholds for *App*^*NL-G-F/NL-G-F*^, and *vice versa*). The conventional threshold analysis was carried out as a control analysis for the DNN analysis. Figure 3 shows the classification performance of the control analysis. There are three points highlighted. First, the averages of the cross-validated accuracies obtained from the optimum features of the stepwise methods (i.e., asterisks in Figure 3) were higher than those of the significant between-genotype and all features. Similar to the DNN analysis, the stepwise feature-selecting method brought the highest classification performance for the control analysis. Second, more features were selected for the optimum classification performances in the control analysis (Figures 2A versus 3A and 2B versus 3B). Third, the variabilities of the optimum classification performances were higher in the control analysis (17.9% versus 9.8% for phase 1, 13.7% versus 0% for phases 1 and 2; Figures 2A versus 3A and 2C versus 3C, respectively). By considering only the averaged accuracies, the optimum performances of DNN and control analyses were relatively comparable (89.3%–100% versus 88.0%–100%). However, the DNN analysis offered steadier performances (i.e., lower variabilities). The DNN analysis also used fewer classifying features, enabling a simplified measurement.

### Compulsivity and learning behaviors influence classification at an early age

Table 2 lists selected features selected by the stepwise method to obtain the optimum classification performance. Parameters from the delay-discounting test of phase 1 dominated the selected features (three out of four features). Meanwhile, three of seven of the selected features of phase 2 came from the PPR learning test. Even though features were optimized from both phases, the features of phase 2 and PPR learning test were still selected. These features suggested the most influence on classification. Furthermore, some of the selected features showed significant between-genotype differences (those marked with asterisks in Table 2). These results support the two between-genotype behavioral differences mentioned above. The early classification (phase 1) made use of the behavioral parameters of the delay-discounting test that revealed early impairment in the *App*^*NL-G-F/NL-G-F*^ mice. In contrast, the later dysfunction of *App*^*NL-G-F/NL-G-F*^ mice in learning flexibility (PPR learning test) characterized the genotype classification in phase 2. The behavioral parameters of the delay-discounting test also showed significant between-genotype differences in phase 2, whereas the behavioral parameters of the PPR learning test exhibited even greater between-genotype differences (two-sample *t* test; *t*_(27)_ = 2.17–4.15 for PPR learning test; *t*_(27)_ = 2.38–3.33 for DD test). Therefore, the selected features were well-grounded with regard to phase-dependent cognitive impairments. The gender was not selected in any classification phases. Thus, similar to the above-stated finding, gender was less able to account for between-genotype differences and classifying genotypes. Table 3 summarizes the selected features obtained from the control analysis. Parameters from the delay-discounting and learning tests were re-selected. Attention-related parameters also dominated the selected features; however, these parameters showed limited between-genotypes differences compared with the parameters of the delay-discounting and learning tests. The optimum feature combination from phase 1 was characterized by high magnitudes for the *App*^*NL-G-F/NL-G-F*^ mice (i.e., *App*^*NL-G-F/NL-G-F*^ > WT), whereas high averaged features of the WT mice determined the classification using features from phase 2 and both phases (i.e., WT > *App*^*NL-G-F/NL-G-F*^). In an optimum feature combination for the control analysis, the selected features presented either *App*^*NL-G-F/NL-G-F*^ > WT or WT > *App*^*NL-G-F/NL-G-F*^ characteristics. Meanwhile, both characteristics (i.e., *App*^*NL-G-F/NL-G-F*^ > WT and WT > *App*^*NL-G-F/NL-G-F*^) could be optimized in the DNN analysis by manipulating the network weights. Features describing the most distinct between-genotype differences were primarily selected and used in the DNN analysis; the number of optimum features for the DNN analysis was thus fewer than that for the control analysis.

**Table 2:** Optimum classification results in the DNN analysis

Test Phase	Accuracy	Description
Phase 1 (N_WT_ = 13, N_*AppNL-G-F/NL-G-F*_ = 14)	89.3% ± 9.8%	1 [Delay-discounting] Lick rate at saccharin bottles during 6-s delay∗ 2 [PP learning] Visit rate to the correct corner chamber on day 1∗ 3 [Delay-discounting] Nose-poke rate at doorways to access the saccharin solution during 1-s delay 4 [Delay-discounting] Nose-poke rate at doorways to access the saccharin solution during 5-s delay∗
Phase 2 (N_WT_ = 14, N_*AppNL-G-F/NL-G-F*_ = 15)	100% ± 0.0%	1 [PPR learning] Visit rate to the correct corner chamber on day 1∗ 2 [Impulsivity] Premature trial rate on day 8 3 [Attention] Correct rate from total non-premature trial at stimulus duration of 0.5 s∗ 4 [PPR learning] Visit rate to the correct corner chamber on day 2∗ 5 [PA learning] Nose-poke error rate during extinction 6 [PP learning] Visit rate to the correct corner chamber on day 4 7 [PPR learning] Visit rate to the correct corner chamber on day 5
Phases 1 and 2 (N_WT_ = 10, N_*AppNL-G-F/NL-G-F*_ = 10)	100% ± 0.0%	1 [PPR learning of phase 2] Visit rate to the correct corner chamber on day 2∗ 2 [Attention of phase 2] Omission error rate at stimulus duration of 0.5 s 3 [PPR learning of phase 2] Visit rate to the correct corner chamber on day 3∗ 4 [PPR learning of phase 1] Visit rate to the correct corner chamber on day 3 5 [PP learning of phase 2] Visit rate to the correct corner chamber on day 4

This table shows optimum classification results (i.e., accuracy) in the DNN analysis and selected features for genotype classification using behavioral data of test tasks during phases 1, 2, and both phases. Asterisks (∗) denote features showing significantly different averages between WT and *App*^*NL-G-F/NL-G-F*^ mice (two-sample *t* test; p < 0.05).

PP, place preference; PPR, place preference reversal; PA, place avoidance.

**Table 3:** Optimum classification results in the control analysis

Test Phase	Accuracy	Description
Phase 1 (N_WT_ = 13, N_*AppNL-G-F/NL-G-F*_ = 14)	88.0 ± 17.9%	1 [Delay-discounting] Lick rate at saccharin bottles during 7-s delay∗ 2 [Delay-discounting] Nose-poke rate at doorways to access the saccharin solution during 3-s delay∗ 3 [Attention] Omission trial rate at stimulus duration of 0.3 s 4 [Attention] Omission error rate at stimulus duration of 1 s 5 [PA learning] Nose-poke error rate during learning 6 [Delay-discounting] Lick rate at saccharin bottles during 2-s delay∗ 7 [PP learning] Visit rate to the correct corner chamber on day 5 8 [PPR learning] Visit rate to the correct corner chamber on day 4 9 [Impulsivity] Premature trial rate on day 1∗ 10 [Impulsivity] Premature trial rate on day 7 11 [PA learning] Nose-poke error rate during retention
Phase 2 (N_WT_ = 14, N_*AppNL-G-F/NL-G-F*_ = 15)	100% ± 0.0%	1 [PPR learning] Visit rate to the correct corner chamber on day 1∗ 2 [Attention] Correct rate from total trial at stimulus duration of 0.5 s∗ 3 [Attention] Premature trial rate at stimulus duration of 1 s 4 [PA learning] Nose-poke error rate during baseline 5 [PP learning] Visit rate to the correct corner chamber on day 6 6 [Attention] Omission error rate at stimulus duration of 1 s 7 [Attention] Correct rate from total non-premature trial at stimulus duration of 1 s 8 [Impulsivity] Premature trial rate on day 8 9 [PPR learning] Visit rate to the correct corner chamber on day 3∗ 10 [PP learning] Visit rate to the correct corner chamber on day 3 11 [Attention] Correct rate from total trial at stimulus duration of 0.3 s
Phases 1 and 2 (N_WT_ = 10, N_*AppNL-G-F/NL-G-F*_ = 10)	90.0% ± 13.7%	1 [PP learning of phase 1] Visit rate to the correct corner chamber on day 1∗ 2 [PP learning of phase 1] Visit rate to the correct corner chamber on day 2∗ 3 [PP learning of phase 2] Visit rate to the correct corner chamber on day 3

This table shows optimum classification results (i.e., accuracy) in the control analysis and selected features for genotype classification using behavioral data of test tasks during phases 1, 2, and both phases. Asterisks (∗) denote features showing significantly different averages between WT and *App*^*NL-G-F/NL-G-F*^ mice (two-sample *t* test; p < 0.05).

PP, place preference; PPR, place preference reversal; PA, place avoidance.

## Discussion

The applications of machine learning algorithms (including deep learning algorithms) have been previously reported in the studies of wild-type mice, flies, rodents, and birds (Kabra et al., 2013; Valleta et al., 2017; Wang, 2019; Mathis and Mathis, 2020; van Dam et al., 2020). However, to the best of our knowledge, this study is the first application of machine learning to automatic behavioral observations in a study of a diseased mouse model. The application of machine learning brought high classification accuracy between the two mouse genotypes even at the preclinical AD stage. The current findings show the potential of machine learning in support of not only animal models but also prognostic mechanisms and preventive care.

### Insights into applications of machine learning on behavioral data

Several studies have reported applications of machine learning to behavioral performance (e.g., clinical, neuropsychological scores) that attempt to predict AD progression within 3–4 years. Clark et al. (2014, 2016) explored novel ways to extract cognitive parameters based on the verbal fluency test and found that the extracted parameters showed benefits over parameters determined with the random forest algorithm. Additional structural brain parameters did not improve predictions (Clark et al., 2016). Furthermore, the application of support vector machine (SVM) together with the feature-selecting method of recursive feature elimination (RFE) demonstrated high prediction accuracy (Grassi et al., 2018). The RFE method mainly selected performance parameters for memory functions, whereas socio-demographic and physiological (e.g., cardiovascular risk) information were less important predictors. The current study consistently showed the potential of machine learning for determining behavioral parameters equivalent to cognitive functions in a KI mouse model to classify the AD-modeled mice (i.e., a high risk of AD) from the WT mice (i.e., with low risk of AD). The benefits of machine learning approaches over conventional analyses were also confirmed by providing more stable classification performances and more efficient feature inputs. The behavioral parameters were computed in relatively simple ways, such as by the correct and incorrect rates, without any advanced extraction methods. The DNN performed slightly better than the random forest, SVM, and linear regression algorithms (89.3% versus 79.4%–87.4% averaged accuracy) at early classification (phase 1) under consideration of different sample types (mouse model versus human), numbers (<30 versus 60–107), and features (Clark et al., 2014, 2016; Johnson et al., 2014; Grassi et al., 2018). The DNN's later classification (phase 2; 100%) was superior to those of the other machine learning algorithms.

Moreover, the current findings confirmed the importance of feature selection. Using all available or apparently significant parameters (Figure 2) did not improve the classification performance. A similar phenomenon was previously observed (Sutoko et al., 2019). Johnson et al. (2014) considered the stepwise feature-selecting method to be ineffective at explaining non-independent features. The genetic algorithm (GA) was implemented to replace the stepwise feature selection, and features selected by the GA method yielded higher prediction accuracy than features selected by the stepwise method. To date, we could not determine which of stepwise, GA, or other feature-selecting methods is the most suitable method for selecting the behavioral parameters of AD-modeled mice. This issue should be addressed in a future study.

### Dysfunctional compulsivity control and learning capability in the preclinical AD stage

Decline in the memory domain is a prominent characteristic of the clinical AD stage (Scheltens et al., 2016; Winblad et al., 2016). Parameters of memory functions have also been reported as useful predictors for the prodromal AD stage (Tatsuoka et al., 2013; Johnson et al., 2014; Grassi et al., 2018). Furthermore, impaired memory function is sometimes associated with Aβ deposition in patients with MCI (Pike et al., 2007; Doraiswamy et al., 2012; Harrington et al., 2013). In this study, the memory function is interpreted from one of the performed tasks, namely, place avoidance. *App*^*NL-G-F/NL-G-F*^ mice relatively showed similar memory functions to WT mice (parameter-group 3; Figure 1) in both phases. Therefore, from the viewpoint of AD-symptomatic cognition, the *App*^*NL-G-F/NL-G-F*^ mice are in the preclinical stage. However, memory cognition is a complex domain that intertwines attention functions and influences the learning process (Brem et al., 2013). In phase 2 (13–17 month old), *App*^*NL-G-F/NL-G-F*^ mice showed lower learning capability (low correct visit rate; parameter-group 1), attention (low correct rate; parameter-groups 7 and 8), and aversive memory (high nose-poke error rate; parameter-group 9) than WT mice did. Despite the between-genotype differences of attention and learning functions, the impairments of its functions in the *App*^*NL-G-F/NL-G-F*^ mice were relatively insubstantial compared with the abnormalities of compulsive/persistent behaviors.

Although learning flexibility was slightly dysfunctional in phase 1 (8–12 months old), compulsive/persistent behaviors (parameter-groups 10 and 11) were significantly exhibited by *App*^*NL-G-F/NL-G-F*^ mice during the delay-discounting test. Delay-discounting decision-making was previously reported to be controlled by hippocampal NMDA receptors (Masuda et al., 2020). Six-month-old *App*^*NL-G-F/NL-G-F*^ mice showed around 5%–10% amyloidosis area in the hippocampus at phase 1 (Masuda et al., 2016). However, the association between abnormal compulsivity and hippocampal Aβ deposition is still unclear. Even though increased compulsivity was observed in the early age, this characteristic is unlikely to be observed in patients with AD (Mendez et al., 1997; Nyatsanza et al., 2003). We argue two points here. First, the standard clinical and cognitive assessments, such as Clinical Dementia Rating (CDR) (Hughes et al., 1982), Mini-Mental State Examination (MMSE) (Folstein et al., 1975; Vos et al., 2013), and AD Cooperative Study-Preclinical Alzheimer's Cognitive Composite (ADCS-PACC) (Donohue et al., 2014; Sperling et al., 2014), do not comprehensively evaluate the compulsivity domain. Second, persistent behaviors may indicate poor behavioral flexibility underlying the complex domain of reversal learning (Izquierdo and Jentsch, 2012). Therefore, the nature of compulsivity in AD is not well understood and remains equivocal.

The habenular complex dysfunction is a hypothetical pathophysiology of impulsive and compulsive behaviors. A large number of nose-pokes during the delay-discounting test were observed in the transgenic mice with dysfunction (Kobayashi et al., 2013). Compulsive behavior has been frequently linked to psychological issues, such as anxiety. The anxiety domain impairs executive functions, including cognitive flexibility and decision-making (Shields et al., 2016; Park and Moghaddam, 2017). Sakakibara et al. evaluated the anxiety domain in AD-modeled mice (e.g., *App*^*NL/NL*^, *App*^*NL-G-F/NL-G-F*^) using the elevated plus maze (Sakakibara et al., 2018). *App*^*NL-G-F/NL-G-F*^ particularly suppressed anxiety and showed anxiolytic behaviors. In this study, emotional stimulations and burdens (e.g., habitual effects) were minimized and controlled for all genotypes; no medial habenula cells were ablated from any of the mice. Therefore, the compulsive behaviors in the *App*^*NL-G-F/NL-G-F*^ mice were likely triggered by compounding AD-related attributes rather than psychological domains.

Even though the *App*^*NL-G-F/NL-G-F*^ mice significantly showed differences in compulsive/persistent behaviors compared with WT mice in phases 1 and 2, none of the AD clinical characteristics (e.g., tau pathology, severe neuronal loss, and memory-symptomatic cognition) was observed in *App*^*NL-G-F/NL-G-F*^ or other *App* KI mice (Saito et al., 2014; Sasaguri et al., 2017). Therefore, the *App* KI mice were preclinical AD-equivalent models (Sakakibara et al., 2018), and the behavioral differences were hypothetically modest (i.e., subclinical) abnormalities. The current findings emphasize the benefit of identification from an early preclinical stage.

### Future perspectives

Here, we demonstrated the benefits of DNN algorithm and stepwise feature selection to classify AD-modeled mice based on preclinical symptoms. These findings confirm the potential of behaviors as biomarkers for early screening. The advantages of early screening based on only behaviors, without complicated measurements, will significantly improve translatability to and practicability of human studies. Applications of DNN algorithm can be highly versatile. The DNN algorithm can be used for different purposes, such as for monitoring prognostic mechanisms and thriving preventive care. There are still opportunities for further improvements in the computation analysis. In the future, advanced analyses and computational implementations aimed at clinical purposes should be undertaken in both animal and human studies.

### Limitations of the study

There are three limitations that should be addressed in future work. First, the current sample number was small. Different from high inter-individual variability in humans, the characteristics of the *App* KI mice were relatively stable. The current findings should be validated on a large dataset to provide insights into important cognitive domains for identification and machine learning advantages. Furthermore, the translational strategy to human studies should be carefully managed due to the high variability and complex cognitive domains. Second, the stepwise feature selection method is irreversible. Therefore, the effect of a suboptimal selection cascades into the subsequent steps. Even though the current results have been cross-validated and optimized to select influential features, the risk of missed selections could not be avoided. Various feature-selecting methods are available; the efficacy and suitability of those methods on behavioral parameters should be examined. Third, network configurations (e.g., number of hidden layers and nodes) may influence the classification performance. In the current study, the network configurations were not optimized. Therefore, the effect of the network configuration on performance should be addressed in the future.

### Resource availability

#### Lead contact

Further information and requests for resources should be directed to and will be fulfilled by the lead contact, Tsukasa Funane (tsukasa.funane.sb@hitachi.com).

#### Materials availability

This study did not generate new unique reagents.

#### Data and code availability

The readily tested code and data had been prepared in the Supplemental information (Data S1).

## Methods

All methods can be found in the accompanying Transparent methods supplemental file.

## Supplemental information

Document S1. Transparent methods, Figures S1–S4, and Tables S1 and S2

Data S1. Code and data used in this study, related to figure 1

## Footnotes

Supplemental information can be found online at https://doi.org/10.1016/j.isci.2021.102198.
