# Supplementary material for: Early Identification of Alzheimer's Disease in Mouse Models: Application of Deep Neural Network Algorithm to Cognitive Behavioral Parameters
Source: iScience. 2021 Feb 16;24(3):102198. doi: 10.1016/j.isci.2021.102198 (PMC7937558; doi:10.1016/j.isci.2021.102198)
Supplement: Document S1. Transparent methods, Figures S1–S4, and Tables S1 and S2 [file mmc1.pdf]

iScience, Volume *24*

Supplemental information

Early Identification of Alzheimer's Disease in Mouse Models: Application of Deep Neural Network Algorithm to Cognitive Behavioral Parameters

Stephanie Sutoko, Akira Masuda, Akihiko Kandori, Hiroki Sasaguri, Takashi Saito, Takaomi C. Saido, and Tsukasa Funane

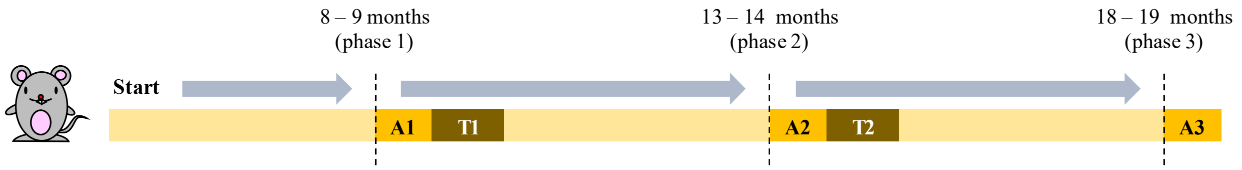

Figure S1. Experimental timeline, Related to Figure 1
Behavioral data were collected during three adaptation phases (A1, A2, and A3) and data on the test (T1 and T2) tasks were collected two times (phases 1 and 2).

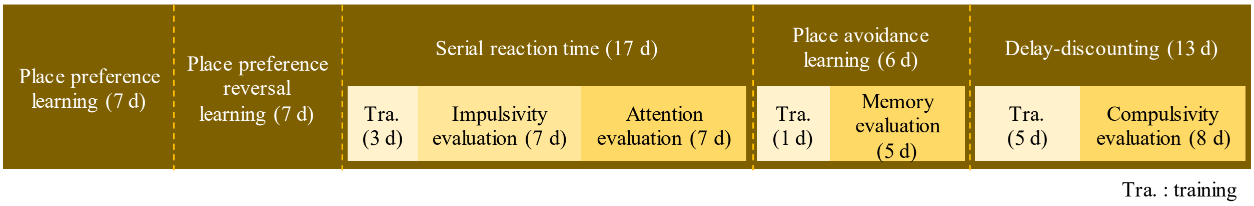

Figure S2. The sequence of test tasks, Related to Figure 1
The test tasks includes place preference learning, place preference reversal learning, serial reaction time, place avoidance learning, and delay-discounting. The test tasks were performed over the course of 50 days (d) in total after the adaptation tasks.

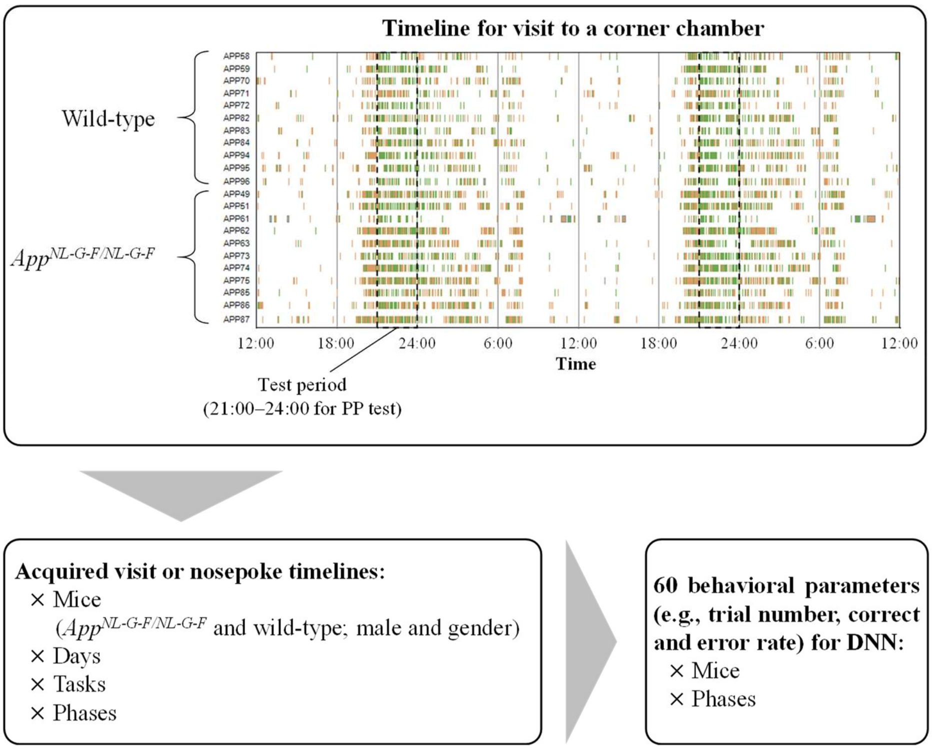

Figure S3. Analytical flow of calculating behavioral parameters from the behavior track, Related to Table 1
An example of visiting data recorded from female mice in the IntelliCage system during the test of place preference (data from only first and second days) are shown. Test period (also analysis target) was 21:00–24:00. Visiting to correct and incorrect corner chambers are displayed by green- and orange-colored horizontal bars.

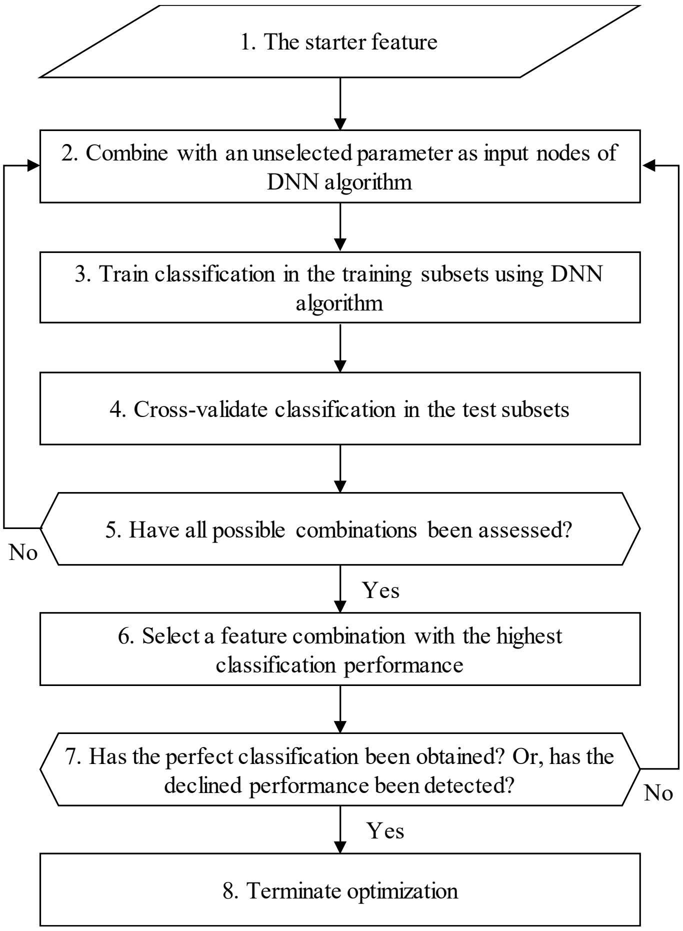

Figure S4. Optimization process for classification analysis, Related to Figure 2
The starter feature (step 1) was defined as the behavioral parameter with the strongest between-genotype difference (the highest *t*-value from a two-sample *t*-test). The starter feature was combined with a feature chosen from among the unselected parameters for the DNN input nodes (step 2). The input nodes were then trained in the training subsets (step 3), and the obtained weights in the hidden layers were cross-validated (5-fold) in the test subsets (step 4). All possible combinations of two features involving the starter feature were assessed (step 5). Among all combinations, the two-feature combination giving the highest classification performance (i.e., highest average test accuracy) was selected (step 6). If the classification performance was found to be 100% accurate or found to decrease dramatically (> 5%) (step 7), the optimization process was terminated (step 8). Otherwise, the optimization process continued to find the best-performing combinations of three, four features, and so on.

Table S1. Behavioral parameters of phase 1 from parameter-groups 1–11, Related to Figure 1 and Table 1

Genotype	Mice ID	Sex	Class 1							Class 2							Class 3							
			D-1	D-2	D-3	D-4	D-5	D-6	D-7	D-1	D-2	D-3	D-4	D-5	D-6	D-7	D-1	D-2	D-3	D-4	D-5	D-6	D-7	D-8
Wild-type	APP10	M	63.09	89.81	79.17	87.04	84.48	93.02	88.68	73.53	88.42	91.82	93.55	91.58	95.10	89.91	75.38	75.68	74.07	57.14	48.39	56.41	34.78	48.39
	APP11	M	60.00	81.82	86.87	79.79	82.80	75.00	70.09	60.77	72.11	82.18	84.17	89.15	92.41	94.83	69.89	65.66	40.63	51.72	37.04	56.67	35.00	50.00
	APP12	M	72.52	87.91	83.18	87.76	87.78	91.09	91.49	68.99	85.71	86.00	86.14	90.82	91.84	100.00	76.22	51.95	17.95	35.71	30.23	16.13	23.81	12.90
	APP22	M	76.13	73.10	89.06	78.23	89.60	92.59	90.83	71.23	79.72	79.55	85.38	86.32	90.00	92.66	77.78	57.75	66.15	61.11	60.98	37.84	47.22	25.93
	APP23	M	–	–	–	–	–	–	–	–	–	–	–	–	–	–	–	–	–	–	–	–	–	–
	APP24	M	89.13	86.92	93.52	90.18	89.81	91.35	94.05	82.27	85.34	88.19	74.51	87.04	90.82	91.23	71.68	41.03	40.82	38.64	15.38	45.71	48.72	37.50
	APP34	M	74.43	87.33	87.73	89.39	87.86	93.79	89.80	82.55	90.06	92.86	91.56	92.81	93.17	92.64	71.36	48.78	18.92	36.36	28.26	20.45	29.55	28.57
	APP35	M	78.95	89.26	83.96	84.85	93.64	94.74	94.29	80.37	87.76	88.89	90.11	90.36	97.20	95.65	71.30	53.85	39.13	17.65	42.86	29.63	58.93	22.22
	APP36	M	80.45	88.33	84.17	82.73	93.22	81.03	85.98	77.11	79.84	86.09	83.06	85.84	90.16	84.75	86.26	78.38	59.46	60.00	50.00	56.41	15.00	30.00
	APP46	M	74.00	93.38	91.67	87.76	83.59	87.86	99.09	75.18	90.91	90.57	81.13	88.89	87.00	73.22	69.32	53.70	32.26	44.44	47.37	36.36	20.59	47.83
	APP48	M	72.12	66.98	81.01	82.65	88.57	88.00	86.75	84.95	85.37	90.38	85.19	82.61	81.52	83.33	80.37	81.65	50.00	63.64	74.51	56.25	69.70	66.67
	APP59	F	63.73	76.25	79.45	77.78	79.41	78.13	84.81	57.66	65.56	68.89	63.33	68.82	80.56	69.86	70.45	68.86	50.94	36.71	50.54	27.91	42.86	40.48
	APP82	F	61.29	75.00	57.14	69.15	74.44	73.75	70.93	56.86	63.16	63.44	68.42	82.91	91.51	89.66	77.86	72.55	43.65	34.53	35.40	43.75	28.87	27.45
	APP83	F	62.94	93.42	93.59	97.46	92.97	94.69	96.49	75.47	94.57	96.10	96.58	95.28	87.84	91.78	68.29	71.29	64.71	47.50	40.00	56.02	41.51	42.19
	APP84	F	55.45	62.50	72.22	80.00	65.33	82.14	73.02	60.47	72.46	69.74	73.33	67.50	83.91	79.27	77.05	80.45	78.07	62.69	40.85	37.50	27.78	24.19
	APP94	F	84.49	90.37	90.45	95.08	91.07	96.35	97.01	82.23	89.19	96.41	99.13	96.27	97.75	96.48	73.55	73.74	43.53	41.89	54.88	50.00	27.03	35.29
	APP96	F	63.12	77.36	81.82	86.87	88.61	85.87	84.16	65.82	81.31	84.54	90.16	91.30	94.59	91.00	65.00	26.25	38.33	29.41	19.61	32.08	28.95	25.93
App$^{NL-G-F/NL-G-F}$	APP01	M	88.95	97.44	92.50	90.41	97.54	93.80	96.05	80.21	88.41	88.89	90.05	90.64	91.55	95.36	53.16	27.55	23.53	36.62	36.17	22.22	20.00	24.66
	APP02	M	60.16	71.59	77.78	71.11	75.27	80.95	75.58	63.27	72.50	77.34	86.46	86.00	91.03	90.38	69.67	66.67	61.04	49.25	34.92	42.31	27.50	32.43
	APP03	M	70.67	84.38	81.65	87.06	96.33	95.33	90.91	73.39	84.44	87.62	89.17	93.86	87.88	97.44	68.13	38.18	34.78	55.32	45.45	42.42	51.85	32.26
	APP13	M	73.53	68.32	82.35	85.14	82.28	79.45	80.95	75.31	72.62	69.57	85.71	73.63	80.52	74.07	75.00	68.89	40.74	6.25	39.13	56.41	34.78	26.67
	APP14	M	–	–	–	–	–	–	–	–	–	–	–	–	–	–	84.71	83.13	73.58	54.55	40.00	29.03	25.93	37.50
	APP15	M	73.21	87.60	81.74	92.78	90.82	92.38	91.75	75.15	87.27	82.93	96.43	93.62	93.50	94.39	71.21	59.04	54.55	59.26	29.73	29.03	38.46	19.35
	APP25	M	71.85	83.33	82.86	88.12	83.17	83.51	95.56	73.60	75.21	83.78	85.86	85.45	93.81	93.94	73.59	69.40	27.91	21.21	40.82	41.67	36.11	32.35
	APP37	M	51.85	63.56	75.54	84.07	87.25	81.98	80.49	67.38	77.42	82.86	88.28	91.74	92.79	90.18	67.02	77.65	43.86	27.91	29.41	42.55	38.64	39.53
	APP38	M	69.18	87.97	89.47	83.62	85.60	94.83	98.25	87.58	90.91	94.21	97.32	97.48	96.63	84.32	64.79	45.54	43.48	25.64	27.78	24.00	34.62	20.93
	APP49	F	48.25	63.33	67.50	75.00	71.23	81.82	80.88	45.16	63.53	68.75	84.29	82.86	98.46	75.00	56.90	61.27	62.36	44.64	36.73	22.58	30.00	23.68
	APP51	F	61.90	66.67	70.00	77.78	70.41	72.38	80.17	49.72	68.60	70.48	89.52	86.11	88.12	88.57	57.31	61.11	30.46	32.00	31.43	31.15	22.22	41.89
	APP61	F	86.27	89.90	97.30	96.63	96.23	95.35	95.74	83.21	94.59	91.20	97.83	96.67	92.31	100.00	76.36	47.62	51.35	14.29	10.53	25.71	44.00	36.36
	APP63	F	47.06	66.67	74.74	75.56	75.34	75.71	69.33	48.54	69.90	68.24	68.81	68.13	64.95	73.03	73.22	57.51	25.22	20.78	29.55	39.29	18.67	43.08
	APP73	F	62.50	69.23	85.00	88.16	73.91	80.60	82.43	48.08	55.42	72.22	70.67	78.31	75.00	86.84	53.33	76.09	77.71	75.51	56.50	44.00	39.06	42.64
	APP74	F	61.54	75.42	91.40	87.10	83.15	85.42	81.52	56.30	78.63	69.77	72.92	79.82	89.87	82.24	62.50	66.93	63.23	48.64	47.07	43.02	34.08	23.66
	APP75	F	51.69	72.41	65.71	65.59	82.50	81.18	63.22	49.15	68.22	70.10	80.28	77.22	84.42	85.33	70.87	49.34	38.05	29.17	40.30	34.78	30.43	35.42
	APP85	F	57.84	65.52	70.69	73.44	74.47	82.00	81.82	49.07	62.11	73.61	78.00	75.00	82.50	94.20	53.33	46.67	38.78	43.24	44.44	36.96	44.90	28.57
	APP86	F	56.41	75.28	80.77	83.82	76.32	84.38	81.67	53.92	80.23	80.36	85.90	89.47	85.37	84.93	83.15	67.24	56.06	34.04	43.18	18.75	39.13	36.36
	APP87	F	46.90	74.23	71.84	81.71	83.53	68.57	73.49	54.95	53.70	65.00	73.74	71.43	74.44	73.26	64.86	40.00	25.58	46.51	37.25	35.09	33.33	36.73

Table S1. Continued

Genotype	Mice ID	Sex	Class 4			Class 5			Class 6			Class 7			Class 8			Class 9			
			0.3 s	0.5 s	1 s	0.3 s	0.5 s	1 s	0.3 s	0.5 s	1 s	0.3 s	0.5 s	1 s	0.3 s	0.5 s	1 s	Baseline	Learning	Retention	Extinction
Wild-type	APP10	M	29.25	30.10	32.52	19.73	6.69	2.80	7.82	7.02	4.55	31.29	39.80	37.41	44.23	56.94	55.44	28.78	1.65	5.31	43.73
	APP11	M	39.27	45.61	41.31	16.83	3.86	0.28	4.62	4.56	4.56	23.43	29.12	31.91	38.59	53.55	54.37	23.93	2.71	3.03	15.64
	APP12	M	20.51	20.07	21.40	23.08	4.28	1.75	11.36	9.21	11.58	38.83	61.51	60.70	48.85	76.95	77.23	25.60	1.69	15.00	37.50
	APP22	M	25.49	24.55	24.73	48.63	10.11	0.73	10.20	8.66	10.91	10.20	46.57	56.73	13.68	61.72	75.36	18.89	1.78	8.16	17.42
	APP23	M	–	–	–	–	–	–	–	–	–	–	–	–	–	–	–	–	–	–	–
	APP24	M	27.12	32.08	30.77	40.52	19.25	1.75	7.52	8.30	6.29	15.03	32.83	45.10	20.63	48.33	65.15	24.49	0.81	1.48	21.08
	APP34	M	24.10	29.80	29.03	31.92	4.97	0.65	7.82	8.28	8.39	26.06	49.67	52.26	34.33	70.75	73.64	24.75	1.75	5.26	43.12
	APP35	M	30.16	31.87	35.62	31.35	9.96	1.37	5.56	7.17	8.68	25.40	39.84	48.40	36.36	58.48	75.18	19.91	0.94	2.35	8.94
	APP36	M	23.83	27.18	27.16	45.08	15.90	1.23	6.22	5.64	6.79	18.65	50.26	59.26	24.49	69.01	81.36	28.74	2.58	15.97	3.51
	APP46	M	33.43	35.53	32.17	29.11	11.01	0.87	7.49	8.49	7.25	24.78	40.88	50.14	37.23	63.41	73.93	29.53	0.29	6.80	11.76
	APP48	M	34.89	43.75	40.10	22.13	8.85	1.04	6.81	2.60	3.65	35.74	36.98	46.88	54.90	65.74	78.26	18.26	0.58	15.98	53.51
	APP59	F	15.79	25.00	26.76	30.26	2.38	1.41	14.47	14.29	9.86	39.47	58.33	61.97	71.43	70.00	69.84	38.96	1.74	10.29	53.42
	APP82	F	39.72	30.71	34.31	21.28	3.15	1.46	12.06	12.60	10.95	26.95	53.54	53.28	40.43	63.55	60.83	25.97	3.04	15.09	22.37
	APP83	F	32.14	33.13	36.42	17.86	3.75	1.99	14.29	13.13	12.58	35.71	50.00	49.01	52.63	60.15	57.36	30.41	2.40	0.00	2.28
	APP84	F	24.24	36.46	28.41	13.13	10.42	3.41	7.07	8.33	6.82	55.56	44.79	61.36	69.62	55.13	68.35	–	–	–	–
	APP94	F	55.26	47.68	52.53	15.35	16.88	1.56	6.58	11.81	6.23	22.81	23.63	39.69	29.21	33.14	43.04	30.95	3.21	0.00	10.64
	APP96	F	29.87	30.00	37.11	41.56	5.38	0.00	7.14	10.77	10.69	21.43	53.85	52.20	41.77	64.22	58.45	11.11	15.79	0.00	5.84
App$^{NL-G-F/NL-G-F}$	APP01	M	21.14	16.56	17.43	45.99	21.02	1.04	10.37	11.68	11.83	16.05	41.19	61.00	20.35	49.36	73.87	14.98	1.46	3.70	4.00
	APP02	M	39.66	32.20	33.61	29.54	13.61	0.84	6.33	8.62	10.23	14.77	36.51	49.06	24.48	53.85	73.90	19.85	0.65	3.28	14.10
	APP03	M	17.97	22.90	22.15	52.88	3.44	1.01	8.47	12.60	10.40	11.86	56.11	61.07	14.46	72.77	78.45	36.47	5.32	14.38	33.80
	APP13	M	37.86	44.17	45.93	20.16	2.50	0.00	8.23	8.75	8.54	23.87	37.92	39.84	38.41	67.91	73.68	40.19	0.90	0.00	5.30
	APP14	M	24.91	27.40	25.95	33.45	11.03	1.04	7.51	6.76	6.57	26.28	45.55	54.33	35.00	62.75	73.36	–	–	–	–
	APP15	M	33.67	37.99	34.99	26.17	7.86	1.15	3.04	2.18	4.59	20.08	36.68	44.17	30.28	59.15	67.94	20.56	0.62	20.30	31.13
	APP25	M	41.76	41.28	42.98	32.67	12.21	0.00	4.83	6.69	8.19	12.78	31.98	41.52	21.95	54.46	72.82	27.27	2.82	23.89	31.57
	APP37	M	28.34	35.39	28.43	27.88	7.60	0.72	8.53	7.13	7.71	18.89	29.22	38.55	26.37	45.22	53.87	27.77	0.61	3.27	48.12
	APP38	M	18.27	20.58	18.02	46.15	9.00	1.50	9.62	9.32	10.21	18.59	48.87	60.06	22.75	61.54	73.26	10.64	3.03	7.19	22.40
	APP49	F	34.82	40.35	34.58	34.82	13.16	0.93	8.04	6.14	5.61	22.32	40.35	58.88	39.06	50.00	63.00	32.29	5.43	3.92	20.62
	APP51	F	34.97	38.93	35.24	14.69	2.01	0.00	15.38	14.09	13.33	34.97	44.97	51.43	50.00	53.60	59.34	25.43	2.20	0.00	9.79
	APP61	F	28.57	23.08	27.22	19.52	4.52	0.63	1.90	3.62	1.27	50.00	68.78	70.89	63.64	74.88	72.26	36.71	0.55	80.00	60.22
	APP63	F	16.16	24.78	19.15	34.34	7.96	2.13	17.17	21.24	18.09	32.32	46.02	60.64	66.67	65.00	76.00	10.96	2.09	0.00	8.16
	APP73	F	40.16	44.19	51.13	23.77	11.63	0.00	8.20	3.10	8.27	27.87	41.09	40.60	40.96	48.18	44.26	26.00	2.87	12.50	23.21
	APP74	F	52.11	49.12	50.29	4.58	3.82	3.18	40.85	43.82	43.64	2.46	3.24	2.89	4.52	6.18	5.43	24.03	10.24	10.42	32.39
	APP75	F	33.98	27.08	37.25	20.39	13.54	0.98	7.77	11.46	3.92	37.86	47.92	57.84	52.70	63.89	60.82	20.73	5.04	14.68	22.63
	APP85	F	39.20	39.53	38.97	25.60	8.53	4.41	2.40	5.43	4.41	32.80	46.51	52.21	45.56	54.05	57.26	14.44	4.43	13.33	21.30
	APP86	F	27.59	23.89	24.22	41.38	15.93	2.34	8.62	3.54	10.16	22.41	56.64	63.28	44.83	70.33	72.32	25.81	2.79	12.63	9.20
	APP87	F	41.38	42.18	43.05	14.94	6.80	0.00	14.37	6.80	15.23	29.31	44.22	41.72	41.46	51.18	49.22	13.92	5.02	9.34	21.39

Table S1. Continued

Genotype	Mice ID	Sex	Class 10									Class 11									
			0 s	1 s	2 s	3 s	4 s	5 s	6 s	7 s	8 s	0 s	0.1 s	1 s	2 s	3 s	4 s	5 s	6 s	7 s	8 s
Wild-type	APP10	M	92.54	73.78	77.84	38.27	17.78	18.94	12.33	15.45	14.16	98.63	98.45	75.98	80.62	10.64	2.57	1.11	0.32	0.35	0.40
	APP11	M	88.11	88.67	79.23	71.09	56.59	41.18	14.50	25.89	13.46	98.70	99.09	99.09	97.71	89.44	69.69	32.37	7.14	6.45	1.09
	APP12	M	80.15	77.10	65.42	33.04	18.52	16.83	18.18	10.68	12.50	93.09	89.76	97.40	75.65	23.07	2.35	0.00	0.00	0.00	0.00
	APP22	M	85.93	87.57	83.44	49.68	34.07	30.82	25.69	23.08	15.83	89.37	96.54	95.06	86.53	36.67	12.47	8.38	1.60	3.23	0.00
	APP23	M	–	–	–	–	–	–	–	–	–	–	–	–	–	–	–	–	–	–	–
	APP24	M	–	–	–	–	–	–	–	–	–	–	–	–	–	–	–	–	–	–	–
	APP34	M	86.36	89.30	85.88	81.77	80.65	81.29	76.25	77.55	67.72	99.00	99.80	99.10	95.04	90.72	88.01	82.95	72.95	65.29	52.88
	APP35	M	94.39	91.01	73.56	50.42	32.08	30.05	31.52	25.17	32.67	94.34	95.36	94.41	75.50	41.85	17.10	5.72	2.76	3.73	3.17
	APP36	M	91.53	91.30	78.87	77.84	84.38	79.43	66.67	66.43	62.91	99.10	100.00	99.61	96.07	91.07	93.24	61.11	53.10	35.23	20.25
	APP46	M	91.22	89.35	85.90	76.97	70.55	44.08	14.72	17.92	15.79	96.92	100.00	100.00	99.91	100.00	46.70	11.36	0.69	0.00	0.00
	APP48	M	76.74	63.69	58.60	59.59	43.85	36.07	25.56	23.53	22.02	87.97	82.12	37.20	41.44	32.51	17.60	10.66	4.47	4.50	4.93
	APP59	F	85.56	83.15	74.00	72.11	69.94	78.89	29.31	8.63	5.52	99.92	100.00	100.00	98.46	91.98	87.57	68.96	18.58	0.00	0.00
	APP82	F	96.64	88.89	76.64	70.30	46.00	34.12	34.57	30.54	31.41	98.26	100.00	100.00	100.00	93.25	5.26	3.15	0.00	0.00	0.00
	APP83	F	–	–	–	–	–	–	–	–	–	–	–	–	–	–	–	–	–	–	–
	APP84	F	88.97	77.68	63.73	67.06	45.87	13.89	16.35	12.15	6.67	92.45	100.00	99.38	77.85	84.15	25.78	0.00	2.89	0.00	0.00
	APP94	F	85.90	83.33	63.91	66.67	55.73	72.97	52.59	47.83	38.52	97.25	96.00	76.36	63.49	56.56	41.65	44.82	14.71	7.96	1.64
	APP96	F	85.06	85.82	77.61	72.49	71.11	68.75	71.36	71.90	64.86	100.00	100.00	100.00	100.00	100.00	100.00	100.00	100.00	100.00	100.00
App$^{NL-G-F/NL-G-F}$	APP01	M	–	–	–	–	–	–	–	–	–	–	–	–	–	–	–	–	–	–	–
	APP02	M	87.63	83.79	87.06	85.33	75.13	72.45	67.63	56.07	56.22	99.58	99.81	96.12	95.01	91.02	79.46	72.96	51.98	51.30	34.48
	APP03	M	93.63	85.80	88.16	88.08	88.89	94.62	84.52	88.68	88.06	100.00	100.00	100.00	100.00	100.00	100.00	100.00	100.00	99.93	100.00
	APP13	M	89.09	88.80	87.39	84.80	84.48	82.54	73.95	64.15	65.18	99.53	99.25	97.04	100.00	95.19	80.41	87.19	69.32	50.47	60.23
	APP14	M	97.93	92.76	97.60	87.59	92.62	87.50	90.91	89.26	94.00	98.51	99.60	95.97	99.13	94.45	86.73	78.33	74.98	47.62	42.08
	APP15	M	92.98	93.07	76.33	56.10	19.85	14.18	8.33	19.42	14.39	93.65	96.64	93.76	80.88	57.13	10.02	6.67	0.77	8.25	2.17
	APP25	M	95.08	91.98	88.64	86.33	89.39	81.51	81.89	72.44	65.09	98.83	100.00	97.99	99.45	93.13	92.61	79.15	76.76	72.05	52.78
	APP37	M	97.91	95.42	96.88	88.28	91.09	91.80	92.00	95.74	95.68	100.00	100.00	100.00	100.00	99.15	98.88	100.00	100.00	100.00	98.68
	APP38	M	97.17	92.54	89.39	74.70	42.05	41.55	53.76	32.31	30.23	99.16	97.39	89.76	95.40	67.68	24.42	14.49	24.18	7.38	6.55
	APP49	F	93.72	92.65	87.77	88.10	89.51	86.27	89.44	96.27	97.74	100.00	100.00	99.67	100.00	99.77	98.85	99.56	100.00	99.93	100.00
	APP51	F	–	–	–	–	–	–	–	–	–	–	–	–	–	–	–	–	–	–	–
	APP61	F	100.00	99.55	100.00	100.00	99.33	99.42	99.32	100.00	100.00	100.00	100.00	100.00	100.00	100.00	100.00	100.00	100.00	100.00	100.00
	APP63	F	–	–	–	–	–	–	–	–	–	–	–	–	–	–	–	–	–	–	–
	APP73	F	77.05	82.84	66.42	71.74	66.47	57.14	84.35	62.75	51.28	98.73	99.93	99.90	96.53	99.10	98.58	98.44	99.63	53.69	25.77
	APP74	F	–	–	–	–	–	–	–	–	–	–	–	–	–	–	–	–	–	–	–
	APP75	F	80.40	72.82	65.56	67.11	57.23	50.99	33.83	22.96	18.32	93.24	99.76	98.98	94.66	93.01	62.93	63.26	24.72	17.60	8.15
	APP85	F	81.36	77.46	76.60	85.21	75.00	72.55	85.38	78.99	83.49	100.00	99.96	99.69	99.91	98.84	96.28	84.44	92.90	97.22	98.90
	APP86	F	68.34	62.98	57.78	66.67	62.20	62.50	81.25	72.27	83.21	100.00	100.00	100.00	100.00	100.00	99.90	99.92	100.00	100.00	100.00
	APP87	F	73.76	71.63	70.68	78.31	51.74	40.00	29.45	21.48	25.68	99.21	99.44	99.14	99.06	95.13	56.72	17.77	8.47	9.19	4.41

Table S2. Behavioral parameters of phase 2 from parameter-groups 1–11, Related to Figure 1 and Table 1

Genotype	Mice ID	Sex	Class 1							Class 2							Class 3							
			D-1	D-2	D-3	D-4	D-5	D-6	D-7	D-1	D-2	D-3	D-4	D-5	D-6	D-7	D-1	D-2	D-3	D-4	D-5	D-6	D-7	D-8
Wild-type	APP10	M	73.21	73.53	85.56	87.80	85.71	84.71	93.42	82.00	91.21	87.23	83.33	88.54	93.67	89.01	74.63	31.58	54.29	42.86	26.32	35.71	39.13	44.44
	APP11	M	69.70	77.78	80.00	88.73	80.95	87.32	90.00	66.32	89.47	92.21	97.10	90.36	90.32	89.41	72.55	58.70	43.48	42.86	25.00	28.57	19.23	36.84
	APP12	M	–	–	–	–	–	–	–	–	–	–	–	–	–	–	–	–	–	–	–	–	–	–
	APP22	M	74.78	81.06	90.00	92.79	85.85	91.80	91.59	71.20	86.76	88.99	89.47	93.64	93.40	91.67	70.83	44.44	61.29	40.00	40.91	58.14	53.57	60.00
	APP23	M	85.94	86.82	92.55	98.02	95.19	96.91	99.11	78.90	88.89	89.47	100.00	95.74	93.48	93.98	57.83	31.58	30.00	23.08	33.33	0.00	18.18	21.74
	APP24	M	80.17	92.00	91.18	92.45	93.55	90.10	91.58	71.72	76.00	85.71	91.36	92.86	90.54	98.53	51.85	33.33	36.36	28.57	42.86	21.43	0.00	40.00
	APP34	M	63.77	71.59	77.08	91.76	93.75	90.11	92.63	80.65	81.58	90.91	96.40	94.23	93.07	89.89	73.17	38.18	46.67	33.33	35.48	21.05	21.62	14.29
	APP35	M	76.58	90.20	89.74	93.68	93.16	94.44	97.25	88.24	95.97	90.32	92.24	92.37	92.92	92.03	73.08	56.82	50.00	45.16	25.00	45.71	37.50	42.11
	APP36	M	61.79	83.70	82.11	92.31	89.69	86.41	91.40	67.33	75.86	77.11	87.65	86.42	76.88	88.42	66.28	33.33	50.00	26.67	28.57	54.84	52.17	47.37
	APP46	M	72.00	80.67	87.76	83.33	89.42	98.21	93.27	69.36	89.84	91.41	92.97	92.24	96.33	94.78	65.22	26.92	37.50	60.00	34.78	36.00	40.00	23.81
	APP48	M	58.23	72.73	84.62	89.36	89.00	92.31	90.00	89.19	96.84	98.70	94.74	98.86	94.62	98.97	76.00	50.00	14.29	35.29	52.00	22.73	26.32	21.43
	APP59	F	81.52	89.13	82.91	96.12	87.61	95.05	94.00	67.19	85.33	95.06	85.58	93.48	92.41	93.94	52.88	27.94	25.00	27.12	26.09	14.81	30.00	30.30
	APP82	F	68.24	85.89	86.72	91.41	88.54	89.22	90.24	77.40	83.10	90.68	86.00	88.04	89.80	87.36	72.31	52.21	52.98	34.17	39.73	41.25	39.20	41.13
	APP83	F	12.73	82.83	91.33	97.12	95.00	93.70	98.06	69.93	91.57	93.97	96.35	97.12	100.00	95.27	55.07	50.00	44.33	39.66	33.33	26.19	30.16	45.78
	APP84	F	58.42	78.41	82.05	93.24	85.37	95.45	84.42	60.00	83.95	89.04	72.97	89.39	85.00	84.15	72.22	53.91	35.21	17.07	35.29	23.19	17.50	50.00
	APP94	F	69.64	57.95	80.30	84.15	86.05	74.74	87.50	78.57	91.75	92.39	93.33	93.83	91.95	96.55	58.82	54.90	37.80	37.21	28.57	31.15	44.44	35.29
	APP96	F	82.14	87.69	90.68	85.22	91.60	91.89	90.44	73.91	80.77	89.62	84.07	89.69	91.15	85.11	53.13	23.68	31.15	42.37	22.64	25.00	28.57	16.67
$App^{NL\text{-}G\text{-}F/NL\text{-}G\text{-}F}$	APP01	M	78.90	95.00	96.81	98.73	97.80	98.80	96.59	4.41	1.62	3.14	4.17	11.27	93.86	91.23	71.30	68.66	34.69	20.00	3.70	9.30	13.89	25.00
	APP02	M	–	–	–	–	–	–	–	–	–	–	–	–	–	–	–	–	–	–	–	–	–	–
	APP03	M	–	–	–	–	–	–	–	–	–	–	–	–	–	–	–	–	–	–	–	–	–	–
	APP13	M	53.41	67.37	76.12	81.82	89.23	80.28	96.43	70.42	72.73	91.38	98.00	90.20	89.36	94.34	68.04	47.73	32.14	30.77	31.25	31.03	33.33	5.88
	APP14	M	61.70	75.86	88.46	94.52	90.41	86.30	98.53	58.93	84.44	82.50	97.01	92.50	92.65	98.65	80.42	72.09	59.57	62.96	36.36	48.15	42.11	61.36
	APP15	M	74.38	77.60	92.55	91.67	96.49	93.75	96.04	75.86	87.61	89.52	91.26	97.96	98.99	98.90	74.29	55.77	30.23	32.14	19.51	28.13	6.67	21.05
	APP25	M	77.65	83.91	91.04	96.00	94.52	91.30	97.87	64.10	81.01	93.06	84.91	88.89	90.20	98.80	72.00	53.33	48.57	50.00	40.91	64.71	31.58	31.82
	APP37	M	71.52	87.59	94.41	93.02	96.00	95.16	95.83	68.15	83.96	86.76	96.63	99.37	96.98	98.66	60.98	22.22	30.91	24.00	19.61	18.75	16.95	11.11
	APP38	M	–	–	–	–	–	–	–	–	–	–	–	–	–	–	–	–	–	–	–	–	–	–
	APP49	F	66.67	82.69	88.17	82.56	88.31	84.44	90.83	62.39	74.07	76.77	88.76	77.53	83.15	83.56	40.51	23.60	16.46	16.67	13.11	24.42	23.21	22.86
	APP51	F	79.74	88.95	96.32	97.60	97.58	100.00	96.04	45.30	78.57	88.07	84.47	89.62	93.69	96.88	77.32	69.96	56.62	50.65	49.54	44.74	40.48	45.89
	APP61	F	–	–	–	–	–	–	–	–	–	–	–	–	–	–	–	–	–	–	–	–	–	–
	APP63	F	56.12	69.62	78.26	80.00	87.30	91.07	96.55	50.00	75.36	81.25	72.22	74.31	70.65	89.04	35.11	22.22	26.32	20.45	28.85	26.09	30.77	14.29
	APP73	F	60.78	64.15	83.33	81.16	77.14	88.06	83.33	47.69	62.63	69.90	69.86	88.73	90.36	73.40	66.42	46.88	29.52	32.22	41.98	45.10	24.10	47.37
	APP74	F	37.50	73.33	74.53	73.21	68.87	78.72	82.05	40.56	55.00	71.58	69.39	71.29	71.91	73.63	69.11	62.74	41.20	38.21	30.43	30.89	35.79	33.21
	APP75	F	42.86	66.00	75.86	87.50	84.42	88.89	88.61	41.73	73.40	71.88	74.65	80.00	82.67	90.41	45.19	27.16	28.36	20.59	38.46	21.31	24.71	23.73
	APP85	F	60.64	67.95	85.94	70.15	84.48	88.89	95.56	54.02	75.00	82.61	87.30	86.44	92.54	83.12	78.32	74.31	70.23	58.90	37.04	55.10	46.34	33.33
	APP86	F	38.66	68.75	68.18	78.46	72.46	78.46	83.05	62.20	77.94	91.07	83.33	86.44	88.89	83.08	79.92	68.91	38.96	37.78	20.00	31.11	33.33	35.71
	APP87	F	80.95	75.68	87.18	85.00	85.39	87.18	90.79	51.79	71.60	78.87	75.34	77.14	83.08	78.26	59.14	35.62	28.33	17.07	16.22	43.59	34.09	27.91

Table S2. Continued

Genotype	Mice ID	Sex	Class 4			Class 5			Class 6			Class 7			Class 8			Class 9			
			0.3 s	0.5 s	1 s	0.3 s	0.5 s	1 s	0.3 s	0.5 s	1 s	0.3 s	0.5 s	1 s	0.3 s	0.5 s	1 s	Baseline	Learning	Retention	Extinction
Wild-type	APP10	M	26.62	28.22	34.76	35.71	17.79	2.67	3.25	7.36	5.88	20.78	30.67	42.78	25.12	37.10	52.55	18.23	1.84	11.48	8.84
	APP11	M	33.82	37.13	37.21	18.14	4.19	3.49	6.86	5.99	7.56	23.04	28.74	38.95	27.62	36.96	49.71	9.65	2.87	32.71	26.96
	APP12	M	–	–	–	–	–	–	–	–	–	–	–	–	–	–	–	–	–	–	–
	APP22	M	33.33	30.50	38.51	38.02	16.50	1.15	7.29	12.00	9.77	8.85	29.00	41.95	10.71	34.22	53.88	14.07	3.40	14.29	8.80
	APP23	M	16.41	14.91	16.79	51.56	21.93	2.92	14.06	10.53	13.14	14.84	42.98	58.39	17.03	49.45	66.55	31.83	2.54	9.56	32.73
	APP24	M	22.00	25.81	25.00	62.00	24.19	3.13	4.00	9.68	10.94	0.00	40.32	65.63	0.00	69.07	107.69	30.74	2.56	20.73	15.10
	APP34	M	20.38	32.39	23.72	41.40	8.52	1.92	6.37	11.36	11.54	21.02	40.34	54.49	24.15	49.44	64.26	16.44	1.21	12.70	6.72
	APP35	M	27.93	21.62	22.22	33.33	9.01	3.33	10.81	7.21	5.56	18.92	42.34	47.78	25.28	52.59	63.44	13.93	1.05	30.74	24.92
	APP36	M	18.42	19.44	18.68	60.53	22.22	1.10	14.47	4.17	8.79	10.53	47.22	67.03	13.89	64.69	84.35	21.95	2.06	23.71	16.72
	APP46	M	19.73	13.51	19.73	60.54	8.11	0.00	7.48	5.41	6.12	10.20	61.49	69.39	11.79	67.66	80.14	33.58	2.06	21.84	15.85
	APP48	M	21.26	22.31	31.67	30.71	13.22	0.83	7.87	3.31	9.17	34.65	52.07	51.67	41.61	63.84	70.19	12.56	2.39	67.44	12.68
	APP59	F	37.27	36.99	39.01	0.63	10.06	3.66	13.84	7.69	12.80	12.42	27.40	34.04	19.80	43.48	55.81	21.29	0.78	36.36	5.92
	APP82	F	36.04	36.04	36.99	0.63	4.73	0.61	21.38	19.53	18.29	17.26	31.98	30.64	26.98	50.00	48.62	32.90	1.76	18.97	21.67
	APP83	F	27.69	33.64	35.88	0.00	14.20	0.61	11.32	14.79	13.41	14.87	27.27	33.53	20.57	41.10	52.29	26.78	1.14	14.74	52.63
	APP84	F	28.26	23.47	28.57	0.00	8.88	1.83	8.18	8.88	8.54	21.74	38.78	45.92	30.30	50.67	64.29	19.75	1.22	36.96	13.69
	APP94	F	26.62	30.43	28.15	0.00	11.24	0.61	22.01	11.24	15.85	18.83	39.13	51.11	25.66	56.25	71.13	25.83	0.51	14.77	10.40
	APP96	F	27.23	34.03	32.65	0.00	7.69	0.61	11.95	11.83	8.54	16.23	35.08	40.82	22.30	53.17	60.61	14.98	1.42	7.32	1.55
App$^{NL-G-F/NL-G-F}$	APP01	M	14.63	13.79	11.72	56.10	18.10	0.69	12.20	9.48	7.59	17.07	52.59	73.79	19.38	59.68	80.28	38.29	2.52	16.55	2.34
	APP02	M	–	–	–	–	–	–	–	–	–	–	–	–	–	–	–	–	–	–	–
	APP03	M	–	–	–	–	–	–	–	–	–	–	–	–	–	–	–	–	–	–	–
	APP13	M	22.37	36.46	25.00	32.89	14.58	3.00	5.26	11.46	4.00	21.05	34.38	51.00	29.83	55.42	68.00	24.57	2.82	3.00	40.99
	APP14	M	47.01	47.06	52.19	23.11	13.73	1.09	7.57	5.88	6.20	9.56	17.65	22.26	11.77	21.64	27.50	31.55	1.72	26.82	12.49
	APP15	M	26.07	22.22	26.46	44.08	30.99	6.35	6.64	2.34	5.82	13.27	28.65	41.80	15.14	32.94	48.60	14.58	1.52	23.22	15.54
	APP25	M	16.39	23.56	20.88	61.20	40.38	1.65	11.48	15.38	12.09	6.01	19.71	57.69	6.60	22.23	65.17	43.88	2.68	25.53	23.67
	APP37	M	20.39	26.18	18.62	44.31	30.90	3.64	10.98	14.16	12.15	16.86	27.47	55.47	18.33	30.94	59.99	24.82	1.99	8.60	23.88
	APP38	M	–	–	–	–	–	–	–	–	–	–	–	–	–	–	–	–	–	–	–
	APP49	F	27.46	33.33	31.50	0.00	11.83	0.61	16.98	17.16	12.80	13.38	29.71	43.31	18.45	44.57	63.22	22.90	4.59	10.91	16.02
	APP51	F	56.13	55.62	51.27	0.00	17.16	6.10	23.90	18.34	19.51	9.69	10.95	14.93	22.08	24.67	30.64	24.50	3.85	19.30	19.78
	APP61	F	–	–	–	–	–	–	–	–	–	–	–	–	–	–	–	–	–	–	–
	APP63	F	27.87	30.66	24.59	0.00	11.83	0.61	15.09	8.28	7.93	17.21	40.88	50.82	23.86	58.95	67.39	26.82	1.85	24.32	9.18
	APP73	F	28.47	25.58	27.14	1.89	10.65	4.27	16.35	15.98	13.41	21.17	31.01	40.71	29.59	41.67	55.88	21.96	1.78	32.20	25.61
	APP74	F	54.24	52.22	55.27	0.63	0.59	2.44	128.30	116.57	137.20	0.00	0.85	0.18	0.00	1.77	0.41	30.36	6.60	28.45	24.67
	APP75	F	27.01	24.81	24.22	0.00	7.69	1.83	17.61	12.43	17.07	21.90	41.86	39.13	30.00	55.67	51.64	30.04	7.69	30.95	26.79
	APP85	F	47.69	46.34	50.28	0.00	12.43	0.61	5.03	6.51	7.32	8.21	21.95	27.93	15.69	40.91	56.18	11.39	1.36	15.38	17.76
	APP86	F	33.33	34.55	41.34	0.00	21.30	1.83	13.21	4.73	10.98	14.55	24.24	42.46	21.82	37.04	72.38	15.24	2.01	2.63	25.70
	APP87	F	38.82	40.49	34.66	0.00	13.61	0.61	15.72	10.06	15.24	11.18	20.25	37.50	18.27	34.02	57.39	17.38	3.83	23.39	30.25

Table S2. Continued

Genotype	Mice ID	Sex	Class 10									Class 11									
			0 s	1 s	2 s	3 s	4 s	5 s	6 s	7 s	8 s	0 s	0.1 s	1 s	2 s	3 s	4 s	5 s	6 s	7 s	8 s
Wild-type	APP10	M	88.55	99.73	49.56	25.96	14.58	14.05	0.00	0.00	0.00	38.83	55.31	91.68	30.58	25.96	14.58	14.05	0.00	0.00	0.00
	APP11	M	82.70	94.19	46.90	36.14	30.51	30.97	22.95	3.50	1.91	41.69	64.53	33.95	55.47	36.14	30.51	30.97	22.95	3.50	1.91
	APP12	M	–	–	–	–	–	–	–	–	–	–	–	–	–	–	–	–	–	–	–
	APP22	M	–	–	–	–	–	–	–	–	–	–	–	–	–	–	–	–	–	–	–
	APP23	M	61.65	44.59	15.58	16.23	8.48	3.16	7.03	4.61	1.42	41.09	69.78	63.05	52.06	16.23	8.48	3.16	7.03	4.61	1.42
	APP24	M	72.94	91.03	0.79	0.00	0.00	0.00	0.00	0.00	0.00	51.21	99.84	98.63	100.00	0.00	0.00	0.00	0.00	0.00	0.00
	APP34	M	–	–	–	–	–	–	–	–	–	–	–	–	–	–	–	–	–	–	–
	APP35	M	63.35	74.02	1.03	0.18	0.33	0.51	0.00	0.00	0.00	11.30	1.94	67.44	0.99	0.18	0.33	0.51	0.00	0.00	0.00
	APP36	M	55.64	57.21	3.72	0.16	0.36	0.00	0.00	1.44	0.00	14.31	24.56	19.49	2.97	0.16	0.36	0.00	0.00	1.44	0.00
	APP46	M	27.38	33.36	6.94	0.00	0.12	0.00	0.00	0.00	0.00	24.71	14.90	73.18	0.13	0.00	0.12	0.00	0.00	0.00	0.00
	APP48	M	84.26	97.03	64.48	0.25	0.00	0.38	0.00	0.00	0.00	36.62	39.17	26.25	14.86	0.25	0.00	0.38	0.00	0.00	0.00
	APP59	F	100.00	100.00	100.00	100.00	100.00	100.00	100.00	94.66	19.87	99.51	100.00	100.00	100.00	100.00	100.00	100.00	100.00	94.66	19.87
	APP82	F	98.56	8.15	0.00	47.36	67.81	55.76	57.17	25.33	14.47	98.29	32.51	8.15	0.00	47.36	67.81	55.76	57.17	25.33	14.47
	APP83	F	100.00	100.00	0.02	0.00	0.00	0.00	0.00	0.00	0.00	92.29	100.00	100.00	0.02	0.00	0.00	0.00	0.00	0.00	0.00
	APP84	F	93.45	1.60	0.00	13.91	5.34	0.00	1.28	2.00	3.06	91.23	30.01	1.60	0.00	13.91	5.34	0.00	1.28	2.00	3.06
	APP94	F	99.92	93.18	98.44	99.95	99.83	48.62	24.57	1.25	0.00	97.64	95.87	93.18	98.44	99.95	99.83	48.62	24.57	1.25	0.00
	APP96	F	0.08	0.04	0.00	0.00	0.00	0.00	0.00	0.00	0.00	0.37	0.41	0.04	0.00	0.00	0.00	0.00	0.00	0.00	0.00
App$^{NL-G-F/NL-G-F}$	APP01	M	100.00	100.00	100.00	98.86	99.15	98.46	99.44	97.58	80.67	82.26	100.00	100.00	99.64	98.86	99.15	98.46	99.44	97.58	80.67
	APP02	M	–	–	–	–	–	–	–	–	–	–	–	–	–	–	–	–	–	–	–
	APP03	M	–	–	–	–	–	–	–	–	–	–	–	–	–	–	–	–	–	–	–
	APP13	M	51.66	35.32	3.83	2.23	0.00	0.00	0.00	0.00	0.00	46.83	50.97	22.03	33.25	2.23	0.00	0.00	0.00	0.00	0.00
	APP14	M	14.15	32.53	8.82	0.00	0.00	0.00	0.00	0.00	0.00	7.97	0.00	0.58	0.22	0.00	0.00	0.00	0.00	0.00	0.00
	APP15	M	73.18	79.50	54.19	48.32	26.83	10.15	4.42	0.00	0.00	41.99	99.06	90.94	98.43	48.32	26.83	10.15	4.42	0.00	0.00
	APP25	M	62.77	25.38	21.52	66.58	33.38	16.61	0.00	0.00	0.24	29.38	97.86	76.50	92.90	66.58	33.38	16.61	0.00	0.00	0.24
	APP37	M	16.98	15.67	4.43	0.00	0.00	0.00	0.55	0.33	0.00	40.45	29.40	32.42	3.27	0.00	0.00	0.00	0.55	0.33	0.00
	APP38	M	–	–	–	–	–	–	–	–	–	–	–	–	–	–	–	–	–	–	–
	APP49	F	0.00	2.98	1.82	0.00	0.75	1.09	0.55	1.12	0.87	18.69	9.91	2.98	1.82	0.00	0.75	1.09	0.55	1.12	0.87
	APP51	F	99.43	99.67	100.00	100.00	100.00	100.00	100.00	96.90	91.90	99.68	98.16	99.67	100.00	100.00	100.00	100.00	100.00	96.90	91.90
	APP61	F	–	–	–	–	–	–	–	–	–	–	–	–	–	–	–	–	–	–	–
	APP63	F	99.99	100.00	100.00	100.00	100.00	100.00	100.00	97.43	84.60	99.89	100.00	100.00	100.00	100.00	100.00	100.00	100.00	97.43	84.60
	APP73	F	98.40	14.35	17.81	96.60	96.82	73.66	56.63	40.89	17.75	98.82	34.37	14.35	17.81	96.60	96.82	73.66	56.63	40.89	17.75
	APP74	F	99.86	19.95	22.90	99.82	97.38	99.61	98.48	97.52	87.36	99.88	44.67	19.95	22.90	99.82	97.38	99.61	98.48	97.52	87.36
	APP75	F	99.12	13.02	10.93	74.45	66.96	53.68	50.44	34.33	29.62	98.31	49.39	13.02	10.93	74.45	66.96	53.68	50.44	34.33	29.62
	APP85	F	99.12	89.83	100.00	95.42	87.94	66.87	54.36	41.98	33.79	98.13	51.47	89.83	100.00	95.42	87.94	66.87	54.36	41.98	33.79
	APP86	F	98.52	97.32	99.95	99.62	99.14	98.16	63.66	67.49	62.80	98.84	68.43	97.32	99.95	99.62	99.14	98.16	63.66	67.49	62.80
	APP87	F	99.76	100.00	100.00	98.84	100.00	99.91	98.93	99.63	100.00	99.67	98.11	100.00	100.00	98.84	100.00	99.91	98.93	99.63	100.00

TRANSPARENT METHODS

Experimental design

Behavioral performance findings of these experiments have been previously reported (Masuda et al., 2016). The experimental data were collected from mice of four genotypes: *App*$^{NL/NL}$, *App*$^{NL\text{-}F/NL\text{-}F}$, *App*$^{NL\text{-}G\text{-}F/NL\text{-}G\text{-}F}$, and wild-type (WT) with C57BL/6J background. The first three genotypes express mutated amyloid precursor protein (APP) comparable to the endogenous APP and were generated by using a KI strategy (Saito et al., 2014). These KI genotypes were prepared at the animal facility of the RIKEN Center for Brain Science (CBS); all experiments were conducted at the same facility. Twelve mice (all males/females) of mixed genotypes were co-housed in a cage (26 × 37 × 19 cm) starting from an early age (i.e., 1–2 months). In total, ninety-six mice (12 males and 12 females for each genotype; 8 cages in total) took part in the study. The mice were allowed to freely access water and food. The daytime was set to be 8:00–20:00 with lights on; lights were off during the nighttime (20:00–8:00). The housing conditions are described in detail elsewhere (Masuda et al., 2016). All animal experiments were approved by the institutional animal care and use committee and carried out according to the RIKEN CBS's guidelines for animal experiments.

Data acquisition

Behavioral data were collected in three phases (ages 8–12, 13–17, and 18–19 months) as the mice performed the adaptation and test tasks described later in "Test paradigms". During data acquisition, the mice were moved from the standard cages to the IntelliCage system (NewBehavior AG, TSE Systems, Switzerland; www.newbehavior.com) (Krackow et al., 2010; Voikar et al., 2010; Kobayashi et al., 2013; Lee et al., 2015). The IntelliCage system consisted of a cage (39 × 58 × 21 cm) and four corner chambers. Two water bottles could be accessed in each corner chamber through two doorways which were equipped with ring antennas. The conditions under how and which doorways were opened were controlled by computer (IntelliCagePlus Controller; TSE Systems, Switzerland). In order to track the mice, a radio-frequency identification transponder (Standard Microchip T-VA, DataMars, Switzerland & Troven, USA) was implanted into each mouse. The implantation procedure is described elsewhere (Masuda et al., 2016). Ad libitum feeding and the above-described light timing were maintained as the conditions of the standard cage. The adaptation tasks were always conducted prior to the test tasks in order to minimize or even rule out the effects of the new environment (i.e., the IntelliCage system) on habitual consequences. After completing all the test tasks, the mice were returned to the standard cages.

Test paradigms

The adaptation tasks were carried out three times (i.e., ages 8–12, 13–17, and 18–19 months), and the test tasks were conducted in the first two phases (i.e., ages 8–12 and 13–17 months), as shown in Figure S1. The test procedures are described in detail elsewhere (Masuda et al., 2016). The characteristics of *App*$^{NL\text{-}G\text{-}F/NL\text{-}G\text{-}F}$ mice have been reported to be close to those of human AD with rapid Aβ deposition (Saito et al., 2014). Furthermore, the adaptation tasks are likely associated with the manner of habituation (i.e., standard cage vs. IntelliCage) rather than cognitive functions. Therefore, the behavioral test data of the WT (i.e., control) and

App$^{NL-G-F/NL-G-F}$ genotypes were solely used in this study to understand the specific behavior related to the AD cognitive characteristics of the mouse model. Five test tasks were carried out in the following sequence (Figure S2): (1) place preference (PP) learning test for seven days, (2) place preference reversal (PPR) learning test for seven days, (3) serial reaction time test (SRTT) for 17 days including 3-day training, 7-day impulsivity evaluation, and 7-day attention evaluation in order, (4) place avoidance (PA) learning test for six days including 1-day training in prior, and (5) delay-discounting test for 13 days including 5-day training in prior. The behavioral parameters extracted from PP, PPR, serial reaction time tests were daily quantified within a specific time window (21:00–24:00); PA and delay-discounting tests assessed the behavioral parameters within the 24-h time window. The PP and PPR learning tests allowed us to evaluate the learning of spatial information and learning flexibility in mice. The performances of PP and PPR in the IntelliCage system have been reported to be relatively equivalent to the performance of Morris water maze (Ryan et al., 2013; Lee et al., 2015). The Morris water maze (Morris, 1981), a standard test, has been used to evaluate the index of spatial learning (D'Hooge et al., 2001) linked to damage to the hippocampus (Kraemer et al., 1996). Furthermore, the SRTT assessed two cognitive functions, i.e., impulsivity and attention. Lesions in the regions related to attentional and inhibitory-control functions [e.g., dorsal-ventral medial prefrontal cortex (Maddux et al., 2011), pedunculopontine tegmental nucleus (Inglis et al., 2001), subthalamic nucleus (Baunez et al., 1997), and medial habenular-interpeduncular nucleus (Kobayashi et al., 2013)] were associated with impaired performance of SRTT. The PA learning test evaluated the ability of the mice in aversive spatial memory and extinction learning situations. Hippocampal (Codita et al., 2010; Voikar et al., 2010; Voikar et al., 2018) and amygdala (Knapska et al., 2006) dysfunctions were observed together with the abnormalities of spatial learning and memory. Meanwhile, the delay-discounting test has been used to examine the serotonin and dopamine systems (Winstanley et al., 2005; Kato et al., 2018) in regard to compulsivity/persistence control (Rachlin et al., 1972). All tasks have been frequently performed in the IntelliCage system for monitoring the mice's behaviors [see (Kiryk et al., 2020) for reviews].

Figure S3 shows the analytical flow of calculating the behavioral parameters from the behavior track (e.g., visit data) recorded by the IntelliCage system. The behavior tract was visualized by the horizontal bars of either the visit or the nosepoke interval to a corner chamber with the timepiece information. Numbers of visits and nosepokes on the correct and incorrect corner chambers were displayed by green- and orange-colored bars. From this tract, the visit/nosepoke interval data (in s; correct-incorrect), frequency (per hour or per day; correct-incorrect), and correct rate (per hour or per day) were computed to obtain the behavioral parameters listed in Table 1 and used by the DNN. Behavioral parameters acquired during all tasks from all mice are provided in Tables S1 and S2 below.

Machine learning algorithm

A DNN (Hochreiter et al., 1997) was used for classifying two genotypes (WT and *App*$^{NL-G-F/NL-G-F}$). A DNN includes an input layer, two or more hidden layers, and an output layer. In our case, the input layer consisted of nodes representing the feature (Table 1) used for genotype classification; the output layer consisted of nodes of class (i.e., two genotypes). There were two hidden layers (100 and 40 nodes) between the input and output layers. The DNN algorithm was inspired by the architecture of interconnected neurons (nodes) to transfer

information. A DNN principally connects all nodes from the previous layer to other nodes in the next layers by mathematically manipulating weights for each connection (60×100 + 100×40 + 40×2 = 1080 connections in maximum) and bias values for each node in the hidden and output layers (100 + 40 + 2 = 142 total nodes). The transformation of all connection operations (i.e., nodes×weights) in the previous layers and bias values into output nodes in the next layers is controlled by activations functions. Here, three activation functions were used: hyperbolic tangent (tanh; input layer to 1st hidden layer), rectified linear unit (ReLU; 1st hidden layer to 2nd hidden layer), and normalized exponential (SoftMax; 2nd hidden layer to output layer). The SoftMax activation function yielded the probabilities of two genotypes. Data were classified according to which of the two genotypes had the higher probability. Python 3.6, a programming language, was used to create the DNN algorithm together with open-source libraries of TensorFlow (Google Brain; www.tensorflow.org) and Keras (keras.io). The DNN algorithm ran on a computer equipped with a Linux OS (Ubuntu 16.04 LTS) and two GPUs (11 Gbps; NVIDIA GeForce® GTX 1080Ti).

Behavioral parameters were used as the input nodes of the features. The number of input nodes could be varied from a minimum of two parameters to a maximum of total number of parameters (60 parameters for each phase; Table 1). Because the behavioral parameters were recorded twice, the input nodes could be parameters from phase 1, phase 2, or both. Gender information (binary quantification; 1 for male and 2 for female) might also be able to be used as an input node. Besides using all the parameters as input nodes, two feature-selecting methods were tried, one that used parameters which significantly suggested between-genotype differences (two-sample *t*-test; $p < 0.05$; degree of freedom = 18–27), and another that optimized selections in regard to best-performing classification results (e.g., accuracy). Sex and interaction (genotype × sex variables) effects had been taken into account in the previous report (Masuda et al., 2016). However, this study's aim was to classify the *App*$^{NL\text{-}G\text{-}F/NL\text{-}G\text{-}F}$ mice from the WT mice regardless the sex variable. Therefore, the feature significance was only evaluated on the basis of the genotype variable. Selecting features may avoid the curse of dimensionality (i.e., data sparsity leads to weakened statistical power). Furthermore, a *k*-fold (5-fold) cross-validation was used to minimize overfitting. The feature selection was optimized by following the stepwise-forward approach (Hocking, 1976; Sutoko et al., 2019). This approach added features to be used as input nodes one-by-one on the condition that the added features provided better classification performance (Figure S4). Classification performance was defined as the average accuracy (i.e., true WT and *App*$^{NL\text{-}G\text{-}F/NL\text{-}G\text{-}F}$ mice) of the test subsets. Because the DNN algorithm requires two input nodes at the minimum, the availability of a starter feature is necessary before the selection process. The parameter with the strongest statistical power in evaluating between-genotype differences was chosen as the starter feature (step 1 in Figure S4). The starter feature was combined with an unselected feature (step 2 in Figure S4), and the classification using that combination as the input nodes was trained (step 3 in Figure S4) and validated (step 4 in Figure S4) in the training and test subsets, respectively. All possible combinations of two features involving the starter feature were assessed (step 5 in Figure S4). Among these combinations, the two-feature combination giving the highest classification performance was selected (step 6 in Figure S4). Before reaching perfect classification performance (i.e. 100% accuracy) or detecting a dramatic performance decline (> 5%) (step 7 in Figure S4), the stepwise selection was continuously performed to optimize the best-performing combinations of three, four,

and five or more features. Otherwise, the optimization process was terminated (step 8 in Figure S4). Classification performances obtained from the optimized features, significant between-genotype parameters, and all parameters were compared. The 5-fold cross-validation and stepwise feature-selecting methods were created and run in the Python 3.6 environment. The details of both stepwise selection and DNN algorithm are written in the section of code availability below. The readily tested code and data had been prepared in the supplemental materials.

Besides mouse deaths, inappropriate transponder position, improper data transfer/record, and dramatic changes in the condition of the mice (e.g., atypically inactive) during the test tasks resulted in missing parameters. The DNN algorithm does not allow any analysis of individual data with missing parameters. Therefore, mice data with missing parameters were omitted from the current study, which reduced the sample number.

Control analysis

In order to confirm the usefulness of the DNN method, a control analysis was also conducted. The analysis was performed on the basis of the conventional threshold approach (i.e., mice having behavioral parameters greater than thresholds were classified as $App^{NL\text{-}G\text{-}F/NL\text{-}G\text{-}F}$, and *vice versa*). Feature selection (i.e., stepwise-forward and significant between-genotype) and a 5-fold cross-validation analysis were also carried out. The use of multiple features was feasible; those features would be averaged. In the selection of between-genotype features (two-sample *t*-test; $p < 0.05$; degree of freedom = 18–27), two feature characteristics (i.e., significant $App^{NL\text{-}G\text{-}F/NL\text{-}G\text{-}F}$ > WT and $App^{NL\text{-}G\text{-}F/NL\text{-}G\text{-}F}$ < WT) were extracted. Furthemore, all features were averaged and used as the input of the control analysis in order to evaluate the benefit of feature-selecting methods. The classification performances were compared between the DNN and control analyses.

CODE AVAILABILITY

```
# import required functions
import numpy as np
import math
import csv
import statistics as st
import scipy.stats as stats

from sklearn.model_selection import StratifiedKFold
from keras.models import Sequential
from keras.layers.core import Dense, Activation
from keras.layers.normalization import BatchNormalization
from keras.optimizers import SGD

# import data
# import behavioral parameters from phases 1, 2, or both
```

```
with open('BehavioralData_phase1.txt') as f:
    reader = csv.reader(f,delimiter='¥t')
    X = np.array(list(reader))

# import genotype indices
with open('GenotypicLabels.txt') as f:
    reader = csv.reader(f,delimiter='¥t')
    z = np.array(list(reader))

# Specify indices with missing values
c_=[]
for c in range(0,X.shape[1]):
    dum = np.zeros((X.shape[0],))
    r_=[]
    for r in range(0,X.shape[0]):
        r_.append(math.isnan(float(X[r,c]))) # true for missing values
    res = [i for i, val in enumerate(r_) if val]
    dum[res]=1 # 1: true and 0: false
    c_.append(dum)

# Select sample data without any missing values
X=X[sum(c_)==0,:]
z=z[sum(c_)==0,:]

# characterize the genotypes
y = z[:,1]
y = y.astype(int)
target_names = ['WT','NL-G-F’]

# evaluate the effect of behavioral parameters on genotypes
RES_stat=[]
for ii in range(0,len(X[0])):
    F, p = stats.f_oneway(X[y==0,ii], X[y==1,ii])
    RES_stat.append(F)

# Common settings
seed = 1234
fold_num = 5 # Cross-validation
```

```
#########################################################################################
#####
# Classification analysis

# Randomize sample orders
indices = np.random.permutation(range(len(X)))

# Create training and test subsets
kfold = StratifiedKFold(n_splits=fold_num, shuffle=True, random_state=seed)

# Create the output (0: WT and 1: App^NL-G-F/NL-G-F)
YY=z[:,1]

# Initial setting
ACC=0 # accuracy at the current step (i.e., step 0)
ACC_ref=0 # the highest obtained accuracy
ACC_list=[] # list of averages of resulted accuracies for all steps
ACC_std=[] # list of standard deviations of accuracies across cross-validation for all steps

# DNN setting
n_hidden1 = 100 # number of nodes for the first hidden layer
n_hidden2 = 40 # number of nodes for the second hidden layer
alpha = 0.01 # learning rate for optimizing the network weights
epochs = 200 # interation number of training
batch_size = 10 # split number of training subsets

# Indices of behavioral parameters (i.e., parameters 1–60)
nfeat=np.array(range(0,len(X[0])))

# DNN has to have more than one input
# Initial input from the most significant parameter
featREF=np.argmax(RES_stat)

# Stepwise forward selection
while ACC > (ACC_ref-0.05) or ACC == 1:
    """
    Termination criteria
```

```
 Either declined accuracy about 5% from the highest obtained accuracy or perfect accuracy (100%)
 """

 # Find indices of the unselected parameters
bool_array=np.in1d(nfeat,featREF)
bool_array=nfeat[bool_array==0]

 stpscore=[] # list of averages of resulted accuracies for each combination of parameters
 stpstdscore=[] # list of standard deviations of accuracies across cross-validation for each combination

 # Iteration to combine the selected parameters with the unselected parameters
for IDfeat in range(0,len(bool_array)):
     # Specifiy the inputs
    XX=X[:,np.append(featREF,bool_array[IDfeat])]

    cvscores = [] # list of resulted accuracies across cross-validation

     # Deep Neural Network (DNN) with cross-validation
    for train, test in kfold.split(XX[indices], YY[indices]):
         n_in = len(XX[0]) # number of inputs
         n_out = len(z[0]) # number of outputs

         # Create the network
        model = Sequential()

         # The input layer to the first hidden layer
        model.add(Dense(n_hidden1, input_dim=n_in))
        model.add(BatchNormalization())
        model.add(Activation('tanh'))

         # The first hidden layer to the second hidden layer
        model.add(Dense(n_hidden2))
        model.add(BatchNormalization())
        model.add(Activation('relu'))

         # The second hidden layer to the output layer
        model.add(Dense(n_out))
        model.add(Activation('softmax'))
```

```
            # Compile the network model
            model.compile(loss='categorical_crossentropy', optimizer=SGD(lr=alpha), metrics=['accuracy'])

            # Train the network model with training subsets
            model.fit(XX[train], z[train], epochs=epochs, batch_size=batch_size,verbose=0)

            # Evaluate the network model with test subsets
            loss_and_metrics = model.evaluate(XX[test], z[test])

            cvscores.append(loss_and_metrics[1])

        # averages and standard deviations of resulted accuracies across cross-validation
        stpscore.append(np.mean(cvscores))
        stpstdscore.append(st.stdev(cvscores))

    # Select the combination of parameters giving the highest averaged accuracy
    featREF=np.append(featREF,bool_array[np.argmax(stpscore)])

    # Update the current accuracy
    ACC=stpscore[np.argmax(stpscore)]

    # Update the lists of averages and standard deviations of accuracies step-by-step
    ACC_list.append(ACC)
    ACC_std.append(stpstdscore[np.argmax(stpscore)])

    # Update the highest obtained accuracy from the list of averaged accuracies
    ACC_ref=ACC_list[np.argmax(ACC_list)]

    # Monitor the classification results for each step
    print("Selected parameters:", featREF)
    print("The list of averaged accuracies:", ACC_list)
    print("The list of standard deviation of resulted accuracies:", ACC_std)

##########################################################################################
#####
```

SUPPLEMENTAL REFERENCES

Baunez, C., and Robbins, T.W. (1997). Bilateral lesions of the subthalamic nucleus induce multiple deficits in an attentional task in rats. *Eur J Neurosci* 9(10), 2086-2099.

Codita, A., Gumucio, A., Lannfelt, L., Gellerfors, P., et al. (2010). Impaired behavior of female tg-arcswe app mice in the intellicage: A longitudinal study. *Behav Brain Res* 215(1), 83-94.

Hochreiter, S., and Schmidhuber, J. (1997). Long short-term memory. *Neural Computation* 9(8), 1735-1780.

Hocking, R.R. (1976). The analysis and selection of variables in linear regression. *Biometrics* 32, 1-49.

Inglis, W.L., Olmstead, M.C., and Robbins, T.W. (2001). Selective deficits in attentional performance on the 5-choice serial reaction time task following pedunculopontine tegmental nucleus lesions. *Behav Brain Res* 123(2), 117-131.

Kato, T.M., Kubota-Sakashita, M., Fujimori-Tonou, N., Saitow, F., et al. (2018). *Ant1* mutant mice bridge the mitochondrial and serotonergic dysfunctions in bipolar disorder. *Molecular Psychiatry* 23, 2039-2049.

Knapska, E., Walasek, G., Nikolaev, E., Neuhäusser-Wespy, F., et al. (2006). Differential involvement of the central amygdala in appetitive versus aversive learning. *Learning and Memory* 13(2), 192-200.

Krackow, S., Vannoni, E., A., C., Mohammed, A.H., et al. (2010). Consistent behavioral phenotype differences between inbred mouse strains in the intellicage. *Genes, Brain and Behavior* 9, 722-731.

Kraemer, P.J., Brown, R.W., Baldwin, S.A., and Scheff, S.W. (1996). Validation of a single-day morris water maze procedure used to assess cognitive deficits associated with brain damage. *Brain Research Bulletin* 39(1), 17-22.

Lee, K., Kobayashi, Y., Seo, H., Kwak, J.-H., et al. (2015). Involvement of camp-guanine nucleotide exchange factor ii in hippocampal long-term depression and behavioral flexibility. *Molecular Brain* 8, 38.

Maddux, J.-M., and Holland, P.C. (2011). Effects of dorsal or ventral medial prefrontal cortical lesions on five-choice serial reaction time performance in rats. *Behav Brain Res* 221(1), 63-74.

Morris, R.G.M. (1981). Spatial localization does not require the presence of local cues. *Learning and Motivation* 12, 239-260.

Rachlin, H., and Green, L. (1972). Commitment, choice and self-control. *Journal of the Experimental Analysis of Behavior* 17, 15-22.

Voikar, V., Colacicco, G., Gruber, O., Vannoni, E., et al. (2010). Conditioned response suppression in the intellicage: Assessment of mouse strain difference and effects of hippocampal and striatal lesions on acquisition and retention of memory. *Behav Brain Res* 213(2), 304-312.

Voikar, V., Krackow, S., Lipp, H.-P., Rau, A., et al. (2018). Automated dissection of permanent effects of hippocampal or prefrontal lesions on performance at spatial, working memory and circadian timing tasks of c57bl/6 mice in intellicage. *Behav Brain Res* 352, 8-22.

Winstanley, C.A., Theobald, D.E.H., Dalley, J.W., and Robbins, T.W. (2005). Interactions between serotonin and dopamine in the control of impulsive choice in rats: Therapeutic implications for impulse control disorders. *Neuropsychopharmacology* 30, 669-682.
